# Screening Environmentally Benign Ionic Liquids for CO_2_ Absorption Using Representation Uncertainty-Based Machine Learning

**DOI:** 10.1021/acs.estlett.4c00524

**Published:** 2024-09-10

**Authors:** Shifa Zhong, Yushan Chen, Jibai Li, Thomas Igou, Anyue Xiong, Jian Guan, Zhenhua Dai, Xuanying Cai, Xintong Qu, Yongsheng Chen

**Affiliations:** † Department of Environmental Science, Institute of Eco-Chongming, School of Ecological and Environmental Sciences, East China Normal University, Shanghai 200241, P. R. China; ‡ School of Civil & Environmental Engineering, Georgia Institute of Technology, Atlanta, Georgia 30332, United States; § Fort Richmond Collegiate, Winnipeg, MB R3T 3B3, Canada

**Keywords:** ionic liquids, CO_2_ absorption, uncertainty quantification, QSPR, ensemble model

## Abstract

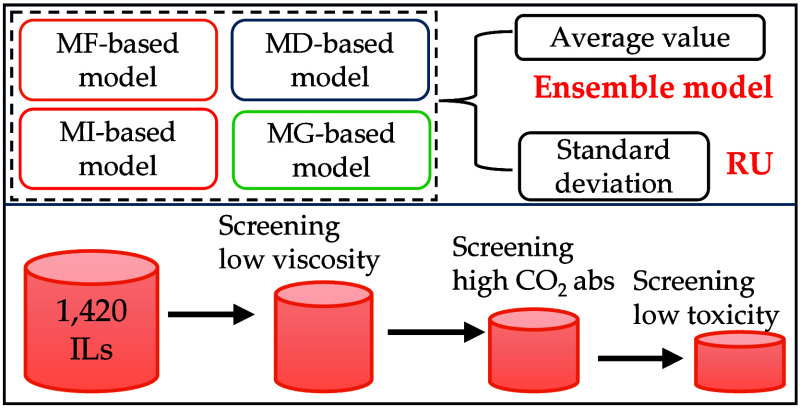

Screening ionic liquids (ILs) with low viscosity, low toxicity, and high CO_2_ absorption using machine learning (ML) models is crucial for mitigating global warming. However, when candidate ILs fall into the extrapolation zone of ML models, predictions may become unreliable, leading to poor decision-making. In this study, we introduce a “representation uncertainty” (RU) approach to quantify prediction uncertainty by employing four IL representations: molecular fingerprint, molecular descriptor, molecular image, and molecular graph. We develop four types of ML models based on these representations and calculate RU as the standard deviation of predictions across these models. Compared to traditional model uncertainty (MU), which is based on hyperparameter variations within a single representation, RU outperforms MU in identifying unreliable predictions across four IL property data sets: viscosity, toxicity, refractive index, and CO_2_ absorption capacity. Furthermore, we develop ensemble models from the four types of models, which show superior predictive performance compared with that of individual models. Using the RU approach, we screened 1420 ILs and identified 37 promising candidates with low viscosity, low toxicity, and high CO_2_ absorption capacity. The predictive performance of our ensemble model, along with the effectiveness of the RU-based approach, was experimentally validated by testing the CO_2_ absorption capacity of 14 ILs. This study not only offers a more reliable method for screening and designing ILs, accelerating the discovery process, but also introduces a new perspective on developing ensemble models with enhanced predictive performance.

## 1 Introduction

Excessive CO_2_ emissions, primarily from fossil fuel combustion, contribute significantly to climate change, presenting grave societal concerns.^1^ One direct solution is capturing CO_2_ from coal-fired plants and other emission sources, but this requires efficient CO_2_ capture–release systems. The widely used amine absorption technology falls short due to its high energy requirements for CO_2_ release and other limitations like thermal degradation, volatility, corrosion, and oxidation.^2−5^ Ionic liquids (ILs), with their low vapor pressure, high thermal stability, and noncorrosive nature, are viewed as promising alternatives.^5−10^ One special characteristic is that their properties can be adjusted by varying the cation–anion combinations. However, this flexibility suggests there could be >10^18^ potential ILs.^11,12^ Given time and resource constraints, it is impractical to experimentally screen each IL for desirable properties, such as high CO_2_ absorption capacity, low toxicity, and low viscosity. A practical approach is to use predictive models to screen the candidate ILs and choose the most promising ones with desirable properties for experimental measurements.

Obtaining the numeric representations of ILs is a prerequisite for developing such predictive models, which should be easily and quickly obtainable. Otherwise, it negates the screening efficiency benefit over experimental measurements. Hence, representations that relied on molecular dynamic simulations, quantum chemical calculations, or density functional theory (DFT) calculations are not suitable due to their time-intensive nature.^13^ Models derived from these representations, such as UNIFAC-IL, COSMO-RS, Soft-SAFT, and regular solution theory (RST), either are restricted to limited-scale screenings or lack sufficient accuracy, often due to oversimplified parameters or assumptions.^10,14−26^

Four readily accessible molecular representations, not relying on time-intensive computational methods, are depicted in Figure S1a: molecular fingerprint (MF), molecular descriptor (MD), molecular image (MI), and molecular graph (MG). These have found applications in developing predictive models within chemistry, drug discovery, and environmental science.^27−36^ Typically, these representations are paired with machine learning (ML) and deep learning (DL) to develop ML- and DL-based models, respectively, distinguishing them from the aforementioned labor-intensive thermodynamic models derived from intricate representations.

One limitation of ML models is their unreliability for samples in extrapolation areas (Text S1), or what is termed “outside of the applicability domain (AD)” in quantitative structure–activity relationship (QSAR) studies. Such unreliability can lead to flawed predictions during screening with chosen ILs potentially offering no advantages over those chosen at random. To counteract this, prior research has developed multiple models by adjusting ML hyperparameters.^14,37−40^ By comparing predictions across these models, researchers can quantify uncertainty using the standard deviation [“model uncertainty” (MU)], allowing for the identification and exclusion of unreliable predictions (Figure S1b).

In our previous study, we employed MF and MD to represent molecules, developing ML models to predict their oxidative rate constants.^41^ We observed predictive performance variations among ML models, which we attributed to the different ways in which these representations captured the features of molecules. As a result, we theorized that utilizing multiple molecular representations, capturing distinct aspects of chemical properties, would yield more robust models for identifying unreliable predictions. We termed this multifaceted approach “representation uncertainty” (RU) (Figure S1c) and hypothesize it to be superior to the MU method that depends on a single molecular representation.

In this study, we integrated four representations (MF, MD, MI, and MG) with ML to develop ML models for four IL properties: CO_2_ absorption capacity, viscosity, toxicity, and refractive index. We then used 10 000 non-IL chemicals as a benchmark to evaluate the efficacy of RU and MU to identify the unreliable predictions (Figure S 1d). Subsequently, we employed RU to methodically screen >1400 ILs, targeting those with low viscosity, high CO_2_ absorption capacity, and low toxicity (Figure S1e). The ensemble model performance and RU-based screened ILs’ CO_2_ absorption capacity were experimentally verified. This research provides a new approach for identifying the unreliable predictions from ML models, with potential applications extending to other molecular fields

## 2 Methods and Materials

### 2.1 Data Sets

A summary of the data sets used in this study is presented in Text S2 and Table S1, among which the refractive index and toxicity data sets were compiled from previous studies,^42−47^ while the viscosity and CO_2_ absorption capacity data sets were manually collected from the ILThermo database (https://ilthermo.boulder.nist.gov/).

### 2.2 Model Development, Model Interpretation, and MU and RU Calculations

The methods for generating MD, MF, MI, and MG representations for ILs and data splitting are illustrated in Text S3 and panels a and b of Figure S2. The root-mean-square error (RMSE), mean absolute error (MAE), and *R*^2^ served as evaluation metrics for evaluating predictive performance. A lower RMSE and a lower MAE paired with a higher *R*^2^ indicate better predictive performance. The details of how to develop MF-, MD-, MG-, and MI-based ML models and their interpretations are illustrated in Text S4. The MU is calculated by four predictions made from four ML models with different hyperparameters using the same representations, which are represented as standard deviations of four predictions made for a queried molecule (Figure S2c). The RU was derived by utilizing models based on MF, MD, MG (trained with optimal hyperparameters), and MI representations. For any given query sample, predictions were made using these four model types. The standard deviation of these predictions was subsequently considered as the RU (Figure S2c).

### 2.3 CO_2_ Absorption Experiments

All chemicals were used without further purification (listed in Text S5). Carbon dioxide gas was purchased from Airgas. The experiments on the CO_2_ absorption capacity with ILs were conducted at atmospheric pressure and 298.15 K. Figure S3 illustrates the system used for CO_2_ absorption.^48^ A typical CO_2_ absorption experiment is illustrated in Text S6.

## 3 Results and Discussion

### 3.1 Comparisons of Model Predictive Performance

**Figure 1:**
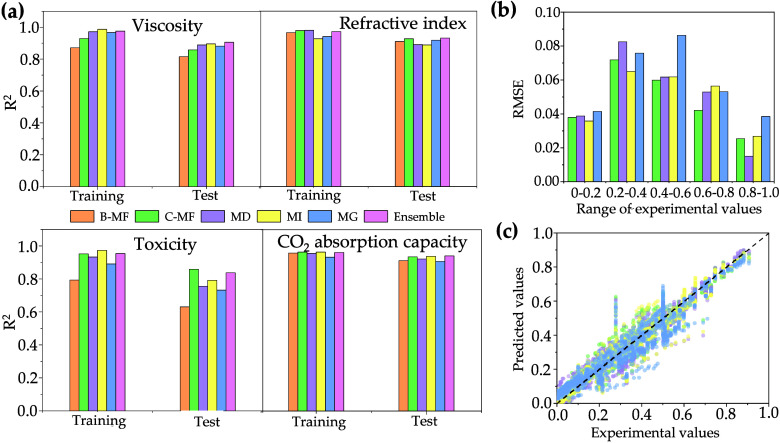
(a) Predictive performance of all developed models for each of the training and test sets from each data set, as evaluated by *R*^2^. (b) Correlations between experimental CO_2_ absorption capacity and predicted values for the CO_2_ absorption data set. (c) Distribution of RMSE in different ranges of experimental CO_2_ absorption capacity.

Figure 1a shows the predictive performance of all developed models for each of the training and test sets from each data set, as evaluated by *R*^2^ (the model performances evaluated by RMSE and MAE are listed in Table S2). Because all models were developed and tested using identical training and test sets, their predictive performance can be directly compared. As anticipated, the predictive performance for training sets consistently surpassed that for the test sets. This is a consequence of models being inherently tailored to training sets. For all data sets, the C-MF-based models outperformed the B-MF-based models in terms of RMSE_test_, MAE_test_, and *R*^2^_test_, indicating the efficacy of C-MF representation, which was consistent with our previous study.^49^ Because B-MF captures the same characteristics of molecules as C-MF but underperforms C-MF in developing accurate predictive models, B-MF is not discussed below.

It is interesting to note the data set-dependent efficacy of representations. MI-based models were superior for the viscosity and CO_2_ adsorption data sets, while C-MF-based models were superior for the refractive index and toxicity data sets. The excellent performance of CNN can be attributed to techniques like transfer learning and data augmentation, strategies frequently employed for data sets with limited size. We then observed that discernible differences arise when these different models predict specific samples, which is evident in the correlation plots (Figure 1b). With the CO_2_ absorption data set as an example, we calculated the RMSE values for different ranges of experimental CO_2_ absorption capacity (Figure 1c). The MI-based model performs better for samples with lower CO_2_ absorption capacity (0–0.4), while C-MF and MD excel at predicting samples with higher CO_2_ absorption capacity (0.6–1). These disparities underscore that each representation captures distinct facets of ILs, which in turn affect the model’s accuracy. Specifically, C-MF encapsulates an IL through a sequence of atomic groups, MD highlights the physicochemical attributes, MI offers a primary representation with relevant features distilled by CNN, and MG amalgamates rudimentary atomic parameters with the IL’s graph structure. Informed by these insights, we developed ensemble models (eq 1), integrating the predictions of these four model types:
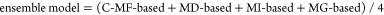
1

The predictive performance (test sets) of ensemble models surpassed the performance of individual models for the viscosity, refractive index, and CO_2_ absorption data sets (Figure 1a).

### 3.2 Comparisons of Model Interpretation

Text S7 and Figure S4 listed the comparison of model interpretations, exemplified by the CO_2_ absorption data set, including global and local interpretations. We here focus on the global interpretation of the C-MF-based model to investigate how top-eight atom groups affect ILs’ properties (Table S3). The same atom groups may contribute to different IL properties in the opposite direction. For example, the F atom group can decrease the log EC_50_ value (i.e., increasing the toxicity) but can increase the CO_2_ absorption capacity. The F atom attached to the quaternary carbon can decrease IL’s viscosity but can increase the CO_2_ absorption capacity. More details about how atomic groups influence the viscosity, toxicity, refractive index, and CO_2_ absorption capacity of ILs are provided in Text S8.

### 3.3 Comparisons between MU and RU in Terms of Identifying Unreliable Predictions

With all of the established models, we can determine both RU and MU. The predictive performances of other three models used for MU calculation on the training set and test set for each data set are listed in Table S4. All of these models also showed satisfactory predictive performance on the test set.

**Figure 2:**
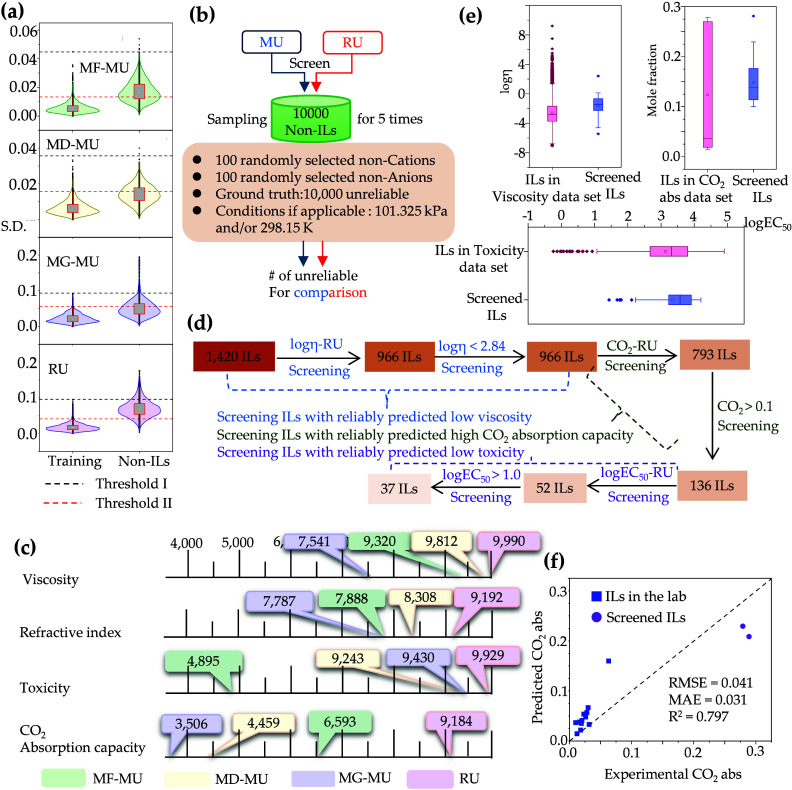
(a) Distributions of the standard deviation (S.D.) for samples in training sets and non-ILs, taking the CO_2_ absorption data set as an example. (b) Work flow that uses MU and RU to discriminate the non-ILs. (c) Average number of identified unreliable predictions by MU and RU using threshold II. (d) Screening ILs with low viscosity, low toxicity, and high CO_2_ absorption capacity by RU. (e) Property ranges of training ILs and the screened ILs. (f) Correlation plot between the experimental CO_2_ absorption capacity and the predicted ones by the ensemble model for 12 ILs in our lab and two screened ILs from 37 screened ILs.

We then examined which type of uncertainty, MU or RU, more effectively identifies unreliable predictions. A threshold value was established to determine prediction reliability on the basis of training uncertainty. With the CO_2_ absorption data set as an example, Figure 2a shows the distribution of the training uncertainty for various MU and RU values. Two thresholds were considered: threshold I (the maximal training uncertainty) and threshold II (the third quartile plus 1.5 times the interquartile range, Q3 + 1.5 × IQR). Threshold I deems all training predictions reliable, while threshold II considers some predictions as outliers and thus unreliable.

We next determined the non-ILs for evaluation of the efficacy of RU and MU in identifying unreliable predictions (Text S9). Five groups of 10 000 non-ILs, assembled from 100 non-cations and 100 non-anions randomly sourced from the ChEMBL database (Figure 2b), were employed to mitigate the risk of random result. If conditions are needed, standard ambient pressure (101.325 kPa) and room temperature (298.15 K) conditions were applied uniformly. We then apply thresholds I and II to quantify the unreliable predictions across these 10 000 non-ILs and finally determined threshold II as the threshold value across all data sets and model types (Text S10).

Figure 2c shows the average number of unreliable predictions for five groups of 10 000 non-ILs identified by MF-based, MD-based, and MG-based MU and RU using threshold II. The specific number of unreliable predictions identified for each group is listed in Table S5. Notably, our introduced RU demonstrated superior effectiveness in identifying unreliable predictions over the other three MUs for all data sets. Stemming from these observations, we will be deploying RU to screen ILs for low viscosity, high CO_2_ absorption capacity, and low toxicity.

### 3.4 Screening ILs with Low Viscosity, Low Toxicity, and High CO_2_ Absorption Capacity by RU

We next set up a pool of 1420 candidate ILs that are from viscosity, toxicity, and refractive index data sets but not tested for CO_2_ absorption capacity. Some ILs are commercially available and not included in all of the data sets used in this study. The screening was executed in a sequential fashion, as depicted in Figure 2d, with candidates in each phase being drawn from the residuals of the previous step. This methodology not only ensures efficiency by concentrating on ILs that meet specific prerequisites but also balances the trade-offs among the properties of CO_2_ absorption capacity, viscosity, and toxicity, thereby saving computational time.

Details of applying RU to screen desirable ILs are illustrated in Text S11. Thirty-seven ILs were identified as having low viscosity, low toxicity, and high CO_2_ absorption potential. Upon closely examining the components of these 37 ILs, we found that they consist of 22 cations and 19 anions (Figure S5). All cations in this study fall under the category of quaternary ammonium compounds, while a majority of anions are based on carbonate, known for their low or nontoxic nature. Previous research has shown that combining these carbonate anions with polymeric ILs based on quaternary ammonium significantly enhances their CO_2_ absorption capabilities, even at ultralow concentrations such as 400 ppm.^50−54^ The molecular structures of these cations and anions are relatively simple, characterized by low molecular weights. This simplicity is advantageous for practical applications, as it contributes to a higher CO_2_ capture efficiency per gram of IL, which is an important metric for real-world usage. In conclusion, our selection of cations and anions aligns well with established domain expertise and research insights.

Figure 2e illustrates the distinctions between the experimental properties of the training ILs and the predicted properties of the screened ILs. The predicted viscosity for the screened ILs fell within the relative top range experimental viscosity of the training ILs. The predicted log EC_50_ for the screened ILs is located at the top range experimental values of the training ILs (i.e., low toxicity). The screened ILs exhibited a predicted CO_2_ absorption capacity of <0.3, which is in the range of the CO_2_ absorption capacity of the training ILs at 298.15 K and 101.325 kPa. Our screening of ILs is based on three criteria (viscosity, toxicity, and CO_2_ absorption capacity) rather than solely considering CO_2_ absorption. Hence, ILs with potentially high CO_2_ absorption capacities may be excluded due to their high viscosity or toxicity. Furthermore, high CO_2_ absorption capacity in ILs is not always advantageous, as the energy consumption of the desorption process must also be considered. High CO_2_ absorption often implies that significant energy will be required for desorption due to the increased absorption enthalpy. Therefore, from the perspectives of energy consumption, toxicity, and viscosity, ILs with moderate CO_2_ absorption capacities may offer a greater practical value. On the contrary, designing ILs with high CO_2_ absorption capabilities and lower absorption enthalpies can be achieved by modifying the anions of the ILs, through the introduction of either electron-withdrawing or electron-donating groups. These modifications alter the strength of the interaction between the anion and the CO_2_. Hence, subsequent modifications based on the stated principle can be applied to optimize the screened ILs’ performance.

### 3.5 Experimental Validation

To validate our ensemble model and RU-based screening method further, we conducted experimental tests on 12 ILs available in our laboratory to measure their CO_2_ absorption. Additionally, we selected two commercially available ILs from the 37 ILs screened above for further testing. Figure 2f presents the correlation plots of the experimental and predicted values. The RMSE, MAE, and *R*^2^ for these 14 ILs were 0.041, 0.031, and 0.797, respectively, comparable to the ensemble model’s predictive performance on the test set (Table S2). Notably, the ILs from our lab exhibited relatively low CO_2_ absorption capacity, whereas those identified through our RU-based approach demonstrated significantly enhanced the CO_2_ absorption capacity.

## 4 Environmental Implications

Our study presents a novel approach to addressing the challenge of identifying unreliable predictions in ML models by emphasizing the importance of diverse IL representations. Instead of relying solely on ML models developed from the same IL representation, which was done in the model uncertainty (MU) approach, we explored ML models built on four different representations. We introduced RU, calculated from these four distinct models, and demonstrated its effectiveness in identifying unreliable predictions, particularly when applied to non-ILs, where predictions are expected to be unreliable. RU proved to be more effective than MU in this regard. Additionally, we developed ensemble models based on these four types of ML models and showed that they offer greater accuracy than individual models, introducing a new perspective on the development of ensemble models.

The computational cost of the RU approach is higher than that of the MU approach because it requires the development of four types of models. However, we believe this is justified as it not only better identifies unreliable predictions but also provides more accurate predictions overall. Finally, we experimentally validated the performance of the ensemble model and the effectiveness of the RU approach by screening ILs with low viscosity, high CO_2_ absorption capacity, and low toxicity. While our research primarily delves into the screening of environmentally friendly ILs for CO_2_ absorption, our methodology holds broader implications across diverse fields from drug discovery to the screening of advanced materials in various industries.

## Data Availability

All of the codes are freely available at GitHub (https://github.com/Shifa-Zhong/CO2_RU.git).

## Supporting Information Available

Details for the results of model interpretation on viscosity, toxicity, and refractive index data sets; node features used for GCNN; candidate hyperparameters for CatBoost and GCNN; selected MDs for MD-based models; top-four best hyperparameters for CatBoost and GCNN models; predictive performances of models developed by different molecular representations for each data set; number of unreliable predictions identified by RU and MU for the other four 10 000 non-ILs; and final selected 37 ILs and their predicted properties (PDF)
The dataset used in this study (XLSX)
The results of screening optimal ML models for MF and MD representations (XLSX)
The hyperparameters obtained by Bayesian optimization for MF-, MD and MG-based ML models (XLSX)
The screened 37 ILs (XLSX)

## References

[ref1] Thuiller W. Climate change and the ecologist. Nature 2007, 448 (7153), 550–552. 10.1038/448550a. 17671497

[ref2] Fisher K. S.; Searcy K.; Rochelle G. T.; Ziaii S.; Schubert C. Advanced amine solvent formulations and process integration for near-term CO_2_ capture success; Trimeric Corp., 2007. 10.2172/945367

[ref3] Kim I.; Svendsen H. F. Heat of absorption of carbon dioxide (CO2) in monoethanolamine (MEA) and 2-(aminoethyl) ethanolamine (AEEA) solutions. Industrial & engineering chemistry research 2007, 46 (17), 5803–5809. 10.1021/ie0616489.

[ref4] Rochelle G. T. Amine scrubbing for CO2 capture. Science 2009, 325 (5948), 1652–1654. 10.1126/science.1176731. 19779188

[ref5] Brennecke J. F.; Gurkan B. E. Ionic liquids for CO2 capture and emission reduction. J. Phys. Chem. Lett. 2010, 1 (24), 3459–3464. 10.1021/jz1014828.

[ref6] Sheldon R. A. Green solvents for sustainable organic synthesis: state of the art. Green Chem. 2005, 7 (5), 267–278. 10.1039/b418069k.

[ref7] Anthony J. L.; Maginn E. J.; Brennecke J. F. Solution thermodynamics of imidazolium-based ionic liquids and water. J. Phys. Chem. B 2001, 105 (44), 10942–10949. 10.1021/jp0112368.

[ref8] Baltus R. E.; Counce R. M.; Culbertson B. H.; Luo H.; DePaoli D. W.; Dai S.; Duckworth D. C. Examination of the potential of ionic liquids for gas separations. Sep. Sci. Technol. 2005, 40 (1–3), 525–541. 10.1081/SS-200042513.

[ref9] Huang J.; Rüther T. Why are ionic liquids attractive for CO2 absorption? An overview. Australian journal of chemistry 2009, 62 (4), 298–308. 10.1071/CH08559.

[ref10] Gurkan B.; Goodrich B.; Mindrup E.; Ficke L.; Massel M.; Seo S.; Senftle T.; Wu H.; Glaser M.; Shah J.; et al. Molecular design of high capacity, low viscosity, chemically tunable ionic liquids for CO2 capture. J. Phys. Chem. Lett. 2010, 1 (24), 3494–3499. 10.1021/jz101533k.

[ref11] Rogers R. D.; Seddon K. R. Ionic liquids--solvents of the future?. Science 2003, 302 (5646), 792–793. 10.1126/science.1090313. 14593156

[ref12] Yan F.; He W.; Jia Q.; Wang Q.; Xia S.; Ma P. Prediction of ionic liquids viscosity at variable temperatures and pressures. Chem. Eng. Sci. 2018, 184, 134–140. 10.1016/j.ces.2018.03.044.

[ref13] Sheridan Q. R.; Schneider W. F.; Maginn E. J. Role of molecular modeling in the development of CO2–reactive ionic liquids. Chem. Rev. 2018, 118 (10), 5242–5260. 10.1021/acs.chemrev.8b00017. 29687999

[ref14] Zhang X.; Wang J.; Song Z.; Zhou T. Data-driven ionic liquid design for CO2 capture: molecular structure optimization and DFT verification. Ind. Eng. Chem. Res. 2021, 60 (27), 9992–10000. 10.1021/acs.iecr.1c01384.

[ref15] Venkatraman V.; Evjen S.; Lethesh K. C.; Raj J. J.; Knuutila H. K.; Fiksdahl A. Rapid, comprehensive screening of ionic liquids towards sustainable applications. Sustainable Energy & Fuels 2019, 3 (10), 2798–2808. 10.1039/C9SE00472F.

[ref16] Zheng W.; Zheng L.; Sun W.; Zhao L. Screening of imidazolium ionic liquids for the isobutane alkylation based on molecular dynamic simulation. Chem. Eng. Sci. 2018, 183, 115–122. 10.1016/j.ces.2018.03.002.

[ref17] Alkhatib I. I.; Ferreira M. L.; Alba C. G.; Bahamon D.; Llovell F. l.; Pereiro A. B.; Araújo J. o. M.; Abu-Zahra M. R.; Vega L. F. Screening of ionic liquids and deep eutectic solvents for physical CO2 absorption by Soft-SAFT using key performance indicators. Journal of Chemical & Engineering Data 2020, 65 (12), 5844–5861. 10.1021/acs.jced.0c00750.

[ref18] Scovazzo P.; Camper D.; Kieft J.; Poshusta J.; Koval C.; Noble R. Regular solution theory and CO2 gas solubility in room-temperature ionic liquids. Industrial & engineering chemistry research 2004, 43 (21), 6855–6860. 10.1021/ie049601f.

[ref19] Liu X.; Zhou T.; Zhang X.; Zhang S.; Liang X.; Gani R.; Kontogeorgis G. M. Application of COSMO-RS and UNIFAC for ionic liquids based gas separation. Chem. Eng. Sci. 2018, 192, 816–828. 10.1016/j.ces.2018.08.002.

[ref20] Wang J.; Song Z.; Cheng H.; Chen L.; Deng L.; Qi Z. Computer-aided design of ionic liquids as absorbent for gas separation exemplified by CO2 capture cases. ACS Sustainable Chem. Eng. 2018, 6 (9), 12025–12035. 10.1021/acssuschemeng.8b02321.

[ref21] Jing G.; Qian Y.; Zhou X.; Lv B.; Zhou Z. Designing and screening of multi-amino-functionalized ionic liquid solution for CO2 capture by quantum chemical simulation. ACS Sustainable Chem. Eng. 2018, 6 (1), 1182–1191. 10.1021/acssuschemeng.7b03467.

[ref22] Peng D.; Picchioni F. Prediction of toxicity of Ionic Liquids based on GC-COSMO method. J. Hazard Mater. 2020, 398, 122964 10.1016/j.jhazmat.2020.122964. 32768829

[ref23] Zhou T.; Shi H.; Ding X.; Zhou Y. Thermodynamic modeling and rational design of ionic liquids for pre-combustion carbon capture. Chem. Eng. Sci. 2021, 229, 116076 10.1016/j.ces.2020.116076.

[ref24] Zhang X.; Liu Z.; Wang W. Screening of ionic liquids to capture CO2 by COSMO-RS and experiments. AlChE J. 2008, 54 (10), 2717–2728. 10.1002/aic.11573.

[ref25] Huang Y.; Zhang X.; Zhang X.; Dong H.; Zhang S. Thermodynamic modeling and assessment of ionic liquid-based CO2 capture processes. Ind. Eng. Chem. Res. 2014, 53 (29), 11805–11817. 10.1021/ie501538e.

[ref26] Wang Y.; Ruan J.; Xue K.; Cheng H.; Zhu Z.; Wang Y.; Zhong L.; Cui P. Phase Equilibrium of p-Xylene and Isobutanol in Ionic Liquid Separations: Thermodynamic and Mechanistic Analysis. ACS Sustainable Chem. Eng. 2024, 12 (8), 3364–3377. 10.1021/acssuschemeng.3c08507.

[ref27] Zhong S.; Zhang K.; Wang D.; Zhang H. Shedding Light On “Black Box” Machine Learning Models for Predicting the Reactivity of HO• Radicals toward Organic Compounds. Chemical Engineering Journal 2021, 405, 126627 10.1016/j.cej.2020.126627.

[ref28] Zhong S.; Hu J.; Yu X.; Zhang H. Molecular image-convolutional neural network (CNN) assisted QSAR models for predicting contaminant reactivity toward OH radicals: Transfer learning, data augmentation and model interpretation. Chemical Engineering Journal 2021, 408, 127998 10.1016/j.cej.2020.127998.

[ref29] Zhong S.; Zhang Y.; Zhang H. Machine Learning-Assisted QSAR Models on Contaminant Reactivity Toward Four Oxidants: Combining Small Data Sets and Knowledge Transfer. Environ. Sci. Technol. 2022, 56, 681–692. 10.1021/acs.est.1c04883. 34908403

[ref30] Zhong S.; Hu J.; Fan X.; Yu X.; Zhang H. A Deep Neural Network Combined with Molecular Fingerprints (DNN-MF) to Develop Predictive Models for Hydroxyl Radical Rate Constants of Water Contaminants. J. Hazard Mater. 2020, 383, 121141–121148. 10.1016/j.jhazmat.2019.121141. 31610411

[ref31] Roszak R.; Beker W.; Molga K.; Grzybowski B. A. Rapid and accurate prediction of p K a values of C–H acids using graph convolutional neural networks. J. Am. Chem. Soc. 2019, 141 (43), 17142–17149. 10.1021/jacs.9b05895. 31633925

[ref32] Goh G. B.; Siegel C.; Vishnu A.; Hodas N. Using rule-based labels for weak supervised learning: a ChemNet for transferable chemical property prediction. In Proceedings of the 24th ACM SIGKDD International Conference on Knowledge Discovery & Data Mining; 2018; pp 302–310.

[ref33] Zhang J.; Wang Q.; Shen W. Message-passing neural network based multi-task deep-learning framework for COSMO-SAC based σ-profile and VCOSMO prediction. Chem. Eng. Sci. 2022, 254, 117624 10.1016/j.ces.2022.117624.

[ref34] Liu J. B. Novel applications of graph theory in chemistry and drug designing. Combinatorial Chemistry & High Throughput Screening 2022, 25 (3), 439–440. 10.2174/1386207325666220104223136. 35038980

[ref35] Sun Y.; Chen M.; Zhao Y.; Zhu Z.; Xing H.; Zhang P.; Zhang X.; Ding Y. Machine learning assisted QSPR model for prediction of ionic liquid’s refractive index and viscosity: The effect of representations of ionic liquid and ensemble model development. J. Mol. Liq. 2021, 333, 115970 10.1016/j.molliq.2021.115970.

[ref36] Kang X.; Zhao Y.; Li J. Predicting refractive index of ionic liquids based on the extreme learning machine (ELM) intelligence algorithm. J. Mol. Liq. 2018, 250, 44–49. 10.1016/j.molliq.2017.11.166.

[ref37] Ma Y.; Guo Z.; Xia B.; Zhang Y.; Liu X.; Yu Y.; Tang N.; Tong X.; Wang M.; Ye X.; et al. Identification of antimicrobial peptides from the human gut microbiome using deep learning. Nat. Biotechnol. 2022, 40, 921–931. 10.1038/s41587-022-01226-0. 35241840

[ref38] Beck B.; Breindl A.; Clark T. QM/NN QSPR models with error estimation: vapor pressure and logP. J. Chem. Inf. Comput. Sci. 2000, 40 (4), 1046–1051. 10.1021/ci990131n. 10955536

[ref39] Chalk A. J.; Beck B.; Clark T. A quantum mechanical/neural net model for boiling points with error estimation. Journal of chemical information and computer sciences 2001, 41 (2), 457–462. 10.1021/ci0004614. 11277737

[ref40] Tetko I. V.; Sushko I.; Pandey A. K.; Zhu H.; Tropsha A.; Papa E.; Oberg T.; Todeschini R.; Fourches D.; Varnek A. Critical assessment of QSAR models of environmental toxicity against Tetrahymena pyriformis: focusing on applicability domain and overfitting by variable selection. J. Chem. Inf. Model. 2008, 48 (9), 1733–1746. 10.1021/ci800151m. 18729318

[ref41] Zhong S.; Zhang Y.; Zhang H. Machine Learning-Assisted QSAR Models on Contaminant Reactivity Toward Four Oxidants: Combining Small Data Sets and Knowledge Transfer. Environ. Sci. Technol. 2022, 56 (1), 681–692. 10.1021/acs.est.1c04883. 34908403

[ref42] Zhao Y.; Huang Y.; Zhang X.; Zhang S. A quantitative prediction of the viscosity of ionic liquids using S σ-profile molecular descriptors. Phys. Chem. Chem. Phys. 2015, 17 (5), 3761–3767. 10.1039/C4CP04712E. 25557501

[ref43] Chen B.-K.; Liang M.-J.; Wu T.-Y.; Wang H. P. A high correlate and simplified QSPR for viscosity of imidazolium-based ionic liquids. Fluid Phase Equilib. 2013, 350, 37–42. 10.1016/j.fluid.2013.04.009.

[ref44] Venkatraman V.; Raj J. J.; Evjen S.; Lethesh K. C.; Fiksdahl A. In silico prediction and experimental verification of ionic liquid refractive indices. J. Mol. Liq. 2018, 264, 563–570. 10.1016/j.molliq.2018.05.067.

[ref45] Sattari M.; Kamari A.; Mohammadi A. H.; Ramjugernath D. A group contribution method for estimating the refractive indices of ionic liquids. J. Mol. Liq. 2014, 200, 410–415. 10.1016/j.molliq.2014.11.005.

[ref46] Wang Z.; Song Z.; Zhou T. Machine learning for ionic liquid toxicity prediction. Processes 2021, 9 (1), 65 10.3390/pr9010065.

[ref47] Venkatraman V.; Alsberg B. K. Predicting CO2 capture of ionic liquids using machine learning. J. Co2 Util 2017, 21, 162–168. 10.1016/j.jcou.2017.06.012.

[ref48] Xiao M.; Liu H.; Gao H.; Olson W.; Liang Z. CO2 capture with hybrid absorbents of low viscosity imidazolium-based ionic liquids and amine. Applied energy 2019, 235, 311–319. 10.1016/j.apenergy.2018.10.103.

[ref49] Zhong S.; Guan X. Count-Based Morgan Fingerprint: A More Efficient and Interpretable Molecular Representation in Developing Machine Learning-Based Predictive Regression Models for Water Contaminants’ Activities and Properties. Environ. Sci. Technol. 2023, 57 (46), 18193–18202. 10.1021/acs.est.3c02198. 37406199

[ref50] Wang T.; Lackner K. S.; Wright A. Moisture Swing Sorbent for Carbon Dioxide Capture from Ambient Air. Environ. Sci. Technol. 2011, 45 (15), 6670–6675. 10.1021/es201180v. 21688825

[ref51] He H.; Zhong M.; Konkolewicz D.; Yacatto K.; Rappold T.; Sugar G.; David N. E.; Matyjaszewski K. Carbon black functionalized with hyperbranched polymers: synthesis, characterization, and application in reversible CO 2 capture. Journal of Materials Chemistry A 2013, 1 (23), 6810–6821. 10.1039/c3ta10699c.

[ref52] He H.; Li W.; Zhong M.; Konkolewicz D.; Wu D.; Yaccato K.; Rappold T.; Sugar G.; David N. E.; Matyjaszewski K. Reversible CO 2 capture with porous polymers using the humidity swing. Energy Environ. Sci. 2013, 6 (2), 488–493. 10.1039/C2EE24139K.

[ref53] He H.; Zhong M.; Konkolewicz D.; Yacatto K.; Rappold T.; Sugar G.; David N. E.; Gelb J.; Kotwal N.; Merkle A.; et al. Three-Dimensionally Ordered Macroporous Polymeric Materials by Colloidal Crystal Templating for Reversible CO2 Capture. Adv. Funct. Mater. 2013, 23 (37), 4720–4728. 10.1002/adfm.201300401.

[ref54] Wang T.; Ge K.; Chen K.; Hou C.; Fang M. Theoretical studies on CO2 capture behavior of quaternary ammonium-based polymeric ionic liquids. Phys. Chem. Chem. Phys. 2016, 18 (18), 13084–13091. 10.1039/C5CP07229H. 27115032

