# Supplementary material for: Screening Environmentally Benign Ionic Liquids for CO2 Absorption Using Representation Uncertainty-Based Machine Learning
Source: Environ Sci Technol Lett. 2024 Sep 10;11(11):1193–9. doi: 10.1021/acs.estlett.4c00524 (PMC11562734; doi:10.1021/acs.estlett.4c00524)
Supplement: Supplementary file 1 — ez4c00524_si_001.pdf [file ez4c00524_si_001.pdf]

**Supporting Information for**

# Screening Environmental-benign Ionic Liquids for $CO_2$ Absorption Using Representation Uncertainty-based Machine Learning

Shifa Zhong [a, #], Yushan Chen [b, #], Jibai Li [a], Thomas Igou [b], Anyue Xiong [c], Jian Guan [a], Zhenhua Dai [a], Xuanying Cai [a], Xintong Qu [a], and Yongsheng Chen [b*]

a Department of Environmental Science, Institute of Eco-Chongming, School of Ecological and Environmental Sciences, East China Normal University, Shanghai 200241, PR China

b School of Civil & Environmental Engineering, Georgia Institute of Technology, Atlanta, Georgia, 30332, United States

c Fort Richmond Collegiate, Winnipeg, MB R3T 3B3, Canada

# These authors contributed to this study equally

***Corresponding Author:** E-mail: yongsheng.chen@ce.gatech.edu

**This file includes:**

Figure S1-S5

Text S1-S11

Tables S1-S10

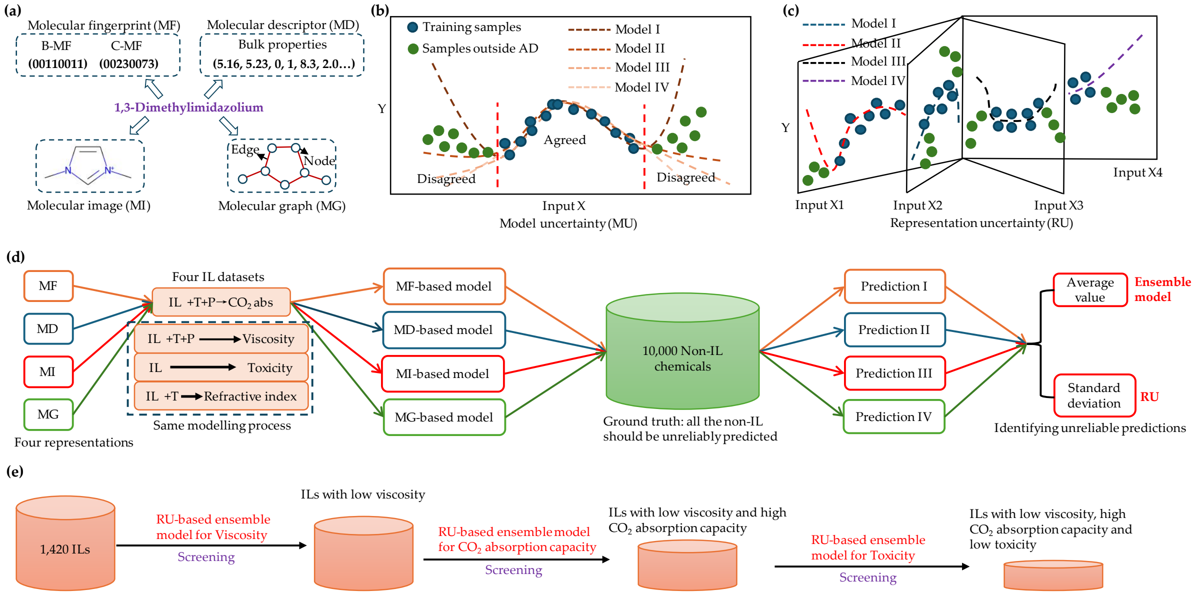


**Figure S1. (a),** Four presentations of one molecule (1,3-Dimethylimidazaolium as an example). (**b**), Previous studies obtained the uncertainty by multiple models developed on the same Input X. (**c**), In this study, we used various representations of Input X (representations) to capture its different characteristics of molecules, based on which we developed multiple representation-based ML models to obtain the uncertainty. **(d),** The scheme of how representation-based ensemble model and RU are obtained. **(e),** The illustration of how to use ensemble model and RU to screen ILs with low viscosity, high $CO_2$ absorption capacity and low toxicity.

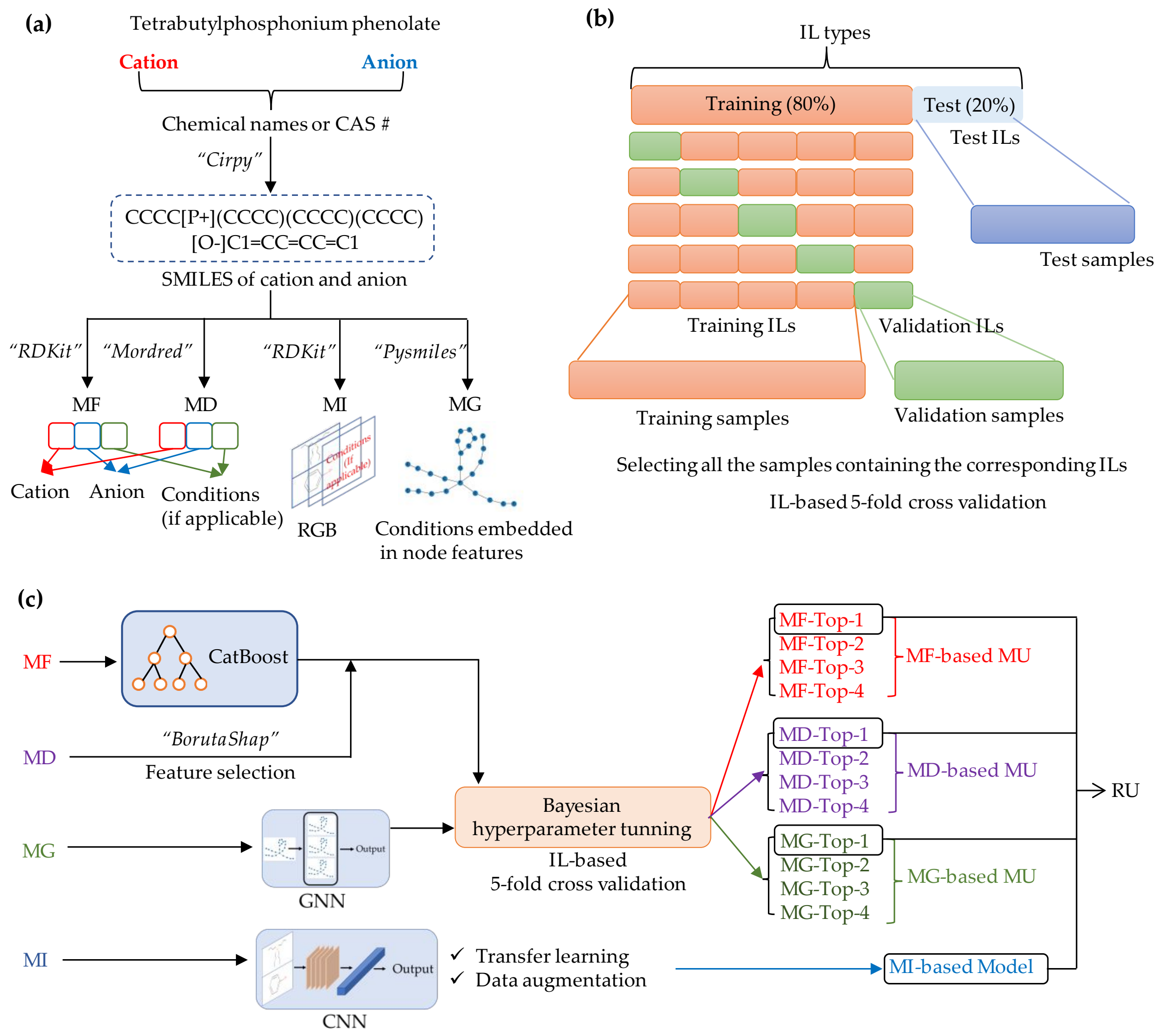


**Figure S2. (a),** the methods to generate MF, MD, MI and MG and to combine conditions if applicable. **(b),** the illustration of IL-based 5-fold cross-validation. **(c),** the model development process and the obtain of MU and RU

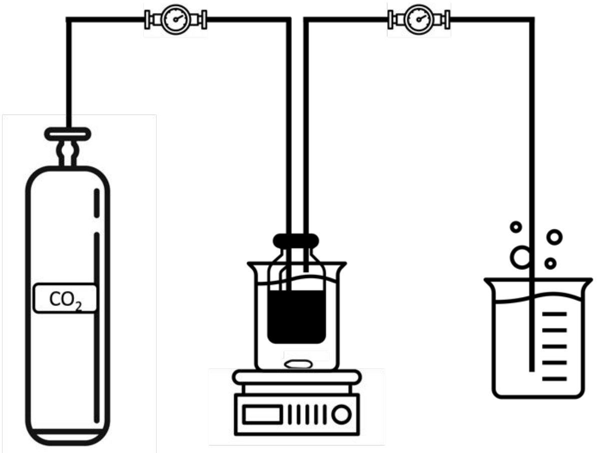


**Figure S3.** Illustration of $CO_2$ absorption system. While ILs are hygroscopic and can absorb moisture, we were not fully aware of the potential presence of other impurities and degradation products in the IL samples at the time of the experiments. Lei et al.'s study indicated that moisture in $CO_2$ can affect the absorption properties of ILs,[1] particularly at the beginning of the $CO_2$ absorption process. However, at the end of the $CO_2$ absorption process, the impact on the amount of $CO_2$ absorbed is minimal. Therefore, this experiment is not expected to be significantly affected by these factors.

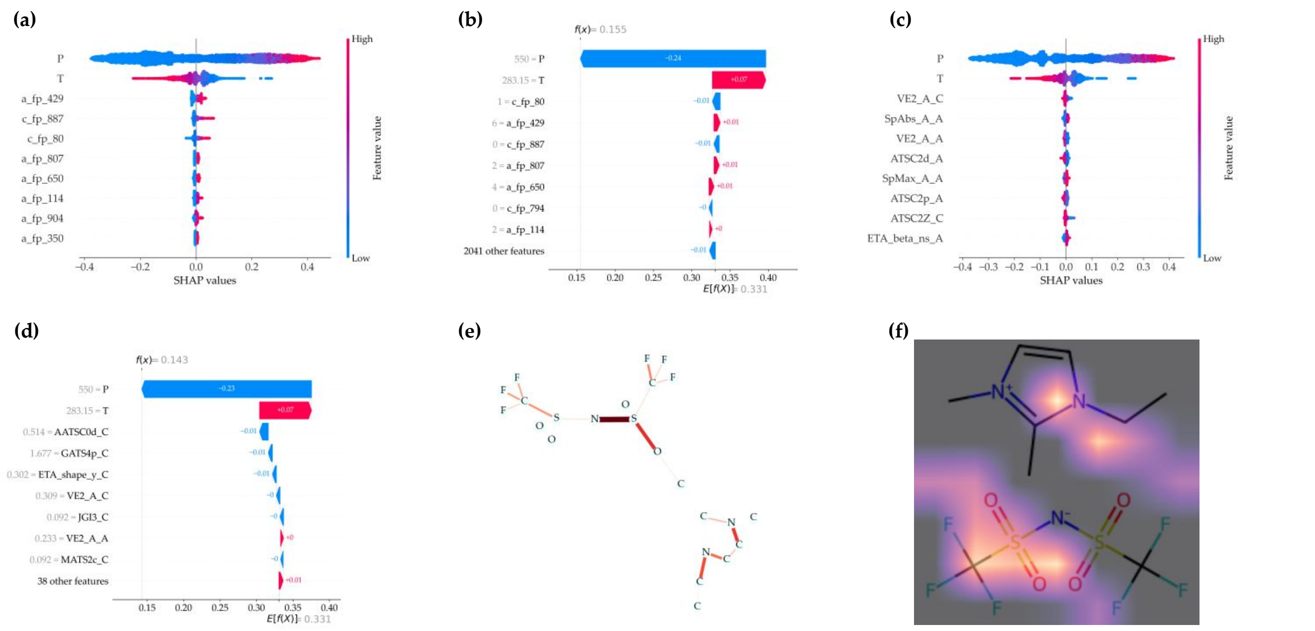


**Figure S4.** The global and local interpretations for C-MF-based (**(a)** and **(b)); **the global and local interpretations for MD-based (**(c)** and **(d)); **the local interpretation for MG-based and MI-based models on prediction of 1-methyl-3-nonylimidazol-1-ium hexafluorophosphate (**(e)** and **(f)**).

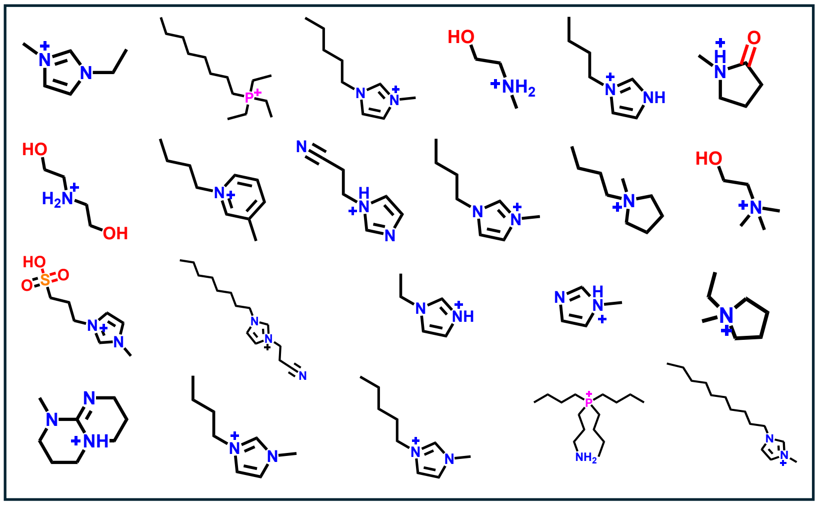

Cation

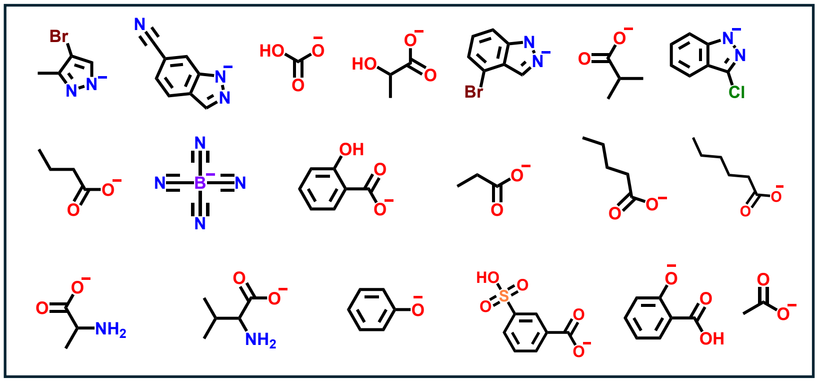

Anion

**Figure S5.** The chemical structures of cations and anions that are composed of the final screened 37 ILs.

## Text S1. The explanation to one limitation of ML models

One limitation of ML models lies in their tendency to produce unreliable predictions for samples that fall within extrapolation areas—regions outside the range of data the model was trained on. This occurs because ML models learn patterns and relationships from the training data, and their ability to generalize to new data is limited by the diversity and scope of that training data. When an ML model encounters a sample

that lies outside the distribution of the training set (an extrapolation), it is forced to predict based on patterns it has not fully learned or encountered before. As a result, the model may generate predictions that are inaccurate or unreliable because it is essentially guessing in uncharted territory. This unreliability in extrapolation areas can lead to poor decision-making, especially in critical applications such as the screening of ionic liquids (ILs) for specific properties where precise predictions are essential.

**Text S2. Description of datasets used in this study**

The toxicity of IL refers to the IL's toxicity against the leukemia rat cell line IPC-81 characterized by $logEC_{50}$ values. The mole fraction that characterizes the $CO_2$ absorption capacity is calculated by **eq. 1**. Refractive index of ILs is less relevant to the $CO_2$ absorption capacity but included in this study to further compare the effectiveness of MU and RU in identifying unreliable predictions. Specifically, by including a property like the refractive index, which is not strongly correlated with $CO_2$ absorption, we aimed to assess how well RU can identify unreliable predictions in a broader context, beyond properties that are directly related to the main focus of the study. In terms of prediction validity, the refractive index's weaker relationship to $CO_2$ absorption allows us to explore the limits of our models' predictive power and the effectiveness of the uncertainty quantification methods. By doing so, we can better understand how different

representations and uncertainty measures perform under varying degrees of relevance to the target property, providing a more comprehensive evaluation of their reliability.

$$\text{mole fraction } (CO_2) = \frac{n_{CO2}}{n_{CO2}+n_{IL}} \quad \textbf{eq. 1}$$

Duplicated samples, defined as identical ILs under the same conditions, were removed. The sample size, types of ILs, cations, anions, and the experimental conditions varied across datasets (**Table S1**), underscoring the diversity inherent within them. More details regarding the types of ILs, ranges of experimental conditions, and objectives are available in "*dataset.xlsx*" in SI.

**Text S3. Generating representations for ILs**

Using tetrabutylphosphonium phenolate as an example, an IL was represented by the simplified molecular-input line-entry system (SMILES) of its cation and anion, which can be obtained by the "CIRpy" Python package If the original datasets lacked them. These SMILES were subsequently canonicalized using the "RDKit" package in Python, which were then used to generate the required representations, as detailed in **Figure S2a**.

**MD**: The MDs for both cations and anions were derived from their canonical SMILES representations using the "Mordred" Python package.[2, 3] An MD is a 1D vector with 1,613 entries, where each represents a unique descriptor. Thorough combining the MDs of cation with anion, an IL is thus presented by a total of 3,226 descriptors. However, not all these descriptors are useful. Initial filtering excluded descriptors with missing,

infinite, or constant values. Subsequent selection was based on a Pearson correlation criterion; when two descriptors had a correlation exceeding 0.9, one was arbitrarily removed. Conditions, when relevant, were appended directly to the MD representation of the IL.

**B-MF and C-MF:** The SMILES for cations and anions were converted into their corresponding B-MF and C-MF using the "RDKit" Python package. The B-MF is a binary representation that encodes atom groups of molecules, while the C-MF further encodes the count information of atom groups. We recently demonstrated that C-MF was more efficient than B-MF in developing more accurate predictive models on different molecular property datasets.[4] Their mechanics and representation have been illustrated in earlier studies.[4-7] Consistent radius and length (1, 1024) were employed for both cations and anions. Like the MD representation, the MF of an IL is represented by combining the MFs of its cation and anion, with conditions attached directly to the IL's MF when relevant.

**MI:** The MIs of ILs were constructed from the MIs of their cations and anions. These images use three channels: red, green, and blue (RGB), each sized 224 x 112 pixels. The amalgamated IL image is 224 x 224 pixels, a dimension that facilitates transfer learning, as many pre-trained models employ this size (https://pytorch.org/vision/stable/models.html). To integrate conditions, the IL image's

channel number was reduced from three to one, and two additional channels filled with conditions were introduced, resulting in an image retaining its three channels but encapsulating both the IL and its conditions.

**MG**: The canonical SMILES of cations and anions were conjoined using a "." symbol, as illustrated by "CCCCP+(CCCC)(CCCC).[O-]C1=CC=CC=C1" for an IL comprising tetrabutylphosphonium and phenolate. The concatenated SMILES of the IL were translated into a NetworkX graph structure using the "pysmiles" package in Python (https://github.com/pckroon/pysmiles). In this structure, nodes symbolize atoms and edges denote bonds. Nodes were characterized by nine atomic features (**Table S6**), in line with Xiong et al's study[8]. While most node features are one-hot encoded, exceptions are the formal charge and radical electron number. Conditions, when relevant, were integrated into each atom's atomic features.

**Data splitting**

For datasets containing experimental conditions — namely Viscosity, Refractive index, and $CO_2$ adsorption capacity (**Table S1**) — they were split into training and test sets based on IL types instead of samples. Splitting based on samples could lead to the same IL type appearing in both training and test sets, risking data leakage.[9] Specifically, ILs were first split into training and test ILs with a ratio of 8:2. Subsequently, all samples associated with the training and test ILs were allocated to the corresponding training or

test sets (**Figure S2b**). This ensured exclusivity: a particular IL type was present either in the training set or the test set, but not both. For 5-fold cross-validation, the same IL-based split was applied to obtain the sub-training and validation sets. The Toxicity dataset, without containing experimental conditions, was randomly split into training and test sets. In all cases, the training set was used during model development, such as feature selection and hyperparameter tuning, while the test set was not exposed to the model and used to evaluate the models' predictive performance.

## Text S4. Developing different representation-based models and their interpretations

### MF- and MD-based model development

For the MF and MD representations, which are attributed to the tabular format, we first screened the optimal ML algorithm among 9 candidate algorithms (**Table S7**) and listed the screened results in *"ML_screening_for_MF_MD.xlsx"* in SI.[6] In the case of MF (B-MF and C-MF), we set the radius and length as (1, 1024) to save the computation time. For MD, we further conducted a feature selection step by the "BorutaShap" and the final number of descriptors and their names used for each datasets were listed in **Table S8**. Because "BorutaShap" is a model-based feature selection method, only the training set was used to conduct feature selection. A detailed working mechanism for "BorutaShap" can refer to this study.[10] The hyperparameters of CatBoost were tuned through Bayesian optimization, incorporating 5-fold cross-validation on the training set.

Bayesian optimization process involves building a probabilistic model, typically a Gaussian process, to approximate the objective function based on past evaluations. This model predicts the function's behavior and guides the search for the optimal input by selecting the next point to evaluate in a way that balances exploration (trying new areas) and exploitation (focusing on promising areas). Bayesian optimization is widely used in machine learning for hyperparameter tuning, where evaluating each set of parameters can be costly. All the optimization results for each hyperparameters are listed in "*BO_hyperopt_MF_MD_MG.xlsx*" in SI. The range for each candidate hyperparameter of the screened ML algorithms is presented in **Table S9**. Upon determining the optimal hyperparameters, the model was retrained on the entire training dataset to finalize the MF-based or MD-based models. Their predictive performance was subsequently evaluated on the test set.

To compute the MU for MF and MD representations, respectively, we utilized the three subsequent best hyperparameter sets (2nd to 4th ranked) to develop three additional MF-based or MD-based models (**Figure S1c**). Consequently, for each dataset, we developed a total of four MF-based and four MD-based models. These models aid in determining the MU, represented as standard deviations of four predictions made for a queried molecule. For each dataset, the top four CatBoost hyperparameters for MF and MD representations can be found in "*BO_hyperopt_MF_MD_MG.xlsx*" in SI.

**MG-based model development**

For the MG representation, the Graph Convolutional Neural Network (GCNN) was employed, given its design tailored to handle graph data formats.[11] The operational intricacies of GCNN are discussed extensively by Kipf and Welling.[11, 12] The hyperparameters of GCNN encompass the number of hidden graph convolution layers, neurons per layer, learning rate, and dropout rate, with the range for each hyperparameter specified in **Table S10**. To counter overfitting, we integrated dropout—a technique that deactivates certain neurons to reduce network complexity,[13] and early stopping, which stops training when the validation score plateaus or diminishes. We confined the candidate number of hidden graph convolutional layers to fewer than 2 due to the relatively compact size of our dataset and observations by Zhou et al., indicating that an excessive number of layers can compromise predictive performance.[14] Likewise, Bayesian optimization was used to tune the their hyperparameters. However, given the early stopping requirement for a validation set, we could not retrain the model solely on the training set. To equitably compare, we developed five models through 5-fold cross-validation and used these collectively to predict the test sets. The final predictions were derived from the averaged values across these five models. Hence, an MG-based model was composed of five models.

In alignment with the methodologies used for MF and MD representations, three sets of suboptimal hyperparameters (“*BO_hyperopt_MF_MD_MG.xlsx*” in SI) were utilized to develop three additional MG-based models (each comprising five models). MU was determined using these MG-based models.

### MI-based model development

For the MI representation, we built upon methodologies from our prior research.[15] In that work, we utilized the DenseNet121—a variant of the Convolutional Neural Network (CNN) algorithm—along with transfer learning and data augmentation techniques.[16] A comprehensive understanding of the workings of CNN, transfer learning, and data augmentation can refer to our study.[15] Given that DenseNet121 comes with predefined hyperparameters, there was no requirement for tuning. Mirroring our approach with the molecular graph (MG) representation, we employed the early stopping technique to counter overfitting during training. Consequently, similar to the MG approach, our MI-based model comprises five models. However, due to the absence of candidate hyperparameters for further MI model development, we did not derive model uncertainty (MU) for the MI representation.

### Model interpretations

The SHapley Additive exPlanation (SHAP) method was employed to interpret the MD-based and MF-based models.[50] This technique had previously been applied for

interpreting MF-based QSAR models targeting •OH radicals and in models predicting membrane performance.[5, 6, 17] For the MIs, we leveraged the gradient-weighted class activation mapping (Grad-CAM) technique to emphasize regions of the molecular images instrumental to model predictions rendered by the CNN.[15, 18] For MGs, we utilized the feature attribution method, pinpointing pivotal atom groupings pertinent to each prediction.[19]

**Text S5. The chemicals used in this study.**

1-Ethyl-3-methylimidazolium tetrafluoroborate (≥98% (HPLC)), 1-Butyl-3-methylimidazolium tetrafluoroborate (≥98%), 1-Hexyl-3-methylimidazolium tetrafluoroborate (≥97.0% (HPLC)), 1-Methyl-3-octylimidazolium tetrafluoroborate (≥97.0% (HPLC)), 1-Decyl-3-methylimidazolium tetrafluoroborate (≥96.5% (HPLC)), 1-Ethyl-3-methylimidazolium hydrogen sulfate (≥95.0%), 1-Ethyl-3-methylimidazolium methyl sulfate (≥98.0% (HPLC)), 1-Ethyl-3-methylimidazolium ethyl sulfate (≥95.0%), 1-Ethyl-3-methylimidazolium trifluoromethanesulfonate (≥95.0% (H-NMR)), 1-Ethyl-3-methylimidazolium bis(trifluoromethyl sulfonyl)imide (≥98.0% (HPLC)), 1-Hexyl-3-methylimidazolium chloride (≥97.0% (HPLC)), Trihexyltetradecylphosphonium chloride (≥95.0% (NMR)) were purchased from Sigma Aldrich, 1-Butyl-3-methylimidazolium

acetate (≥95.0% (HPLC)), 1-Ethyl-3-methylimidazolium acetate (≥94.0% (HPLC)) were purchased from Tokyo Chemical Industry Co., Ltd.

**Text S6. The typical $CO_2$ absorption experiment**

The amount of $CO_2$ absorbed was evaluated by measuring the weight of each bottle, which contained 10 mL of the ionic liquid. $CO_2$ was bubbled through the ILs in a glass container at a flow rate of approximately 100 mL/min. The glass bottle was partially immersed in a water bath maintained at the required temperature. An electronic balance, with an accuracy of ±0.000001 g, measured the amount of $CO_2$ absorbed at regular intervals. The $CO_2$ absorption capacity of ILs was measured by weighing them, and the absorption experiment was continued until the weight of the ILs stopped increasing. To assess the reproducibility of the experiments, we repeated them three times.

**Text S7. The comparisons of model interpretation**

**Figures S4a-f** present interpretations of the four model types, exemplified by the $CO_2$ absorption dataset. Model interpretation is indispensable given the often perceived "black box" nature of ML. Interpreting ML models ensures that predictions are grounded in reasonable logic. Interpretations can be broadly categorized as global or local interpretation. Global interpretation sheds light on the general behavior of a model, revealing how features influence predictions and which features are pivotal or redundant.

Feature importance is a common metric for global interpretation. Local interpretation delves into the reasoning behind specific predictions for individual samples.

For the C-MF and MD representations, the SHAP method facilitates both global and local interpretations. For instance, global interpretations reveal that lower temperatures and higher pressures increase $CO_2$ absorption (**Figures S4a** and **S4b**), which is consistent with the experimental findings. However, the interpretation of C-MF-based model is more easily understood than that of MD-based models because the physicochemical meanings of MDs, such as "VE2_A", "SpAbs_A" and "ATSC2p" (Figure 4B), are difficult to understand. **Figures S4c and S4d** elucidate local interpretation, exemplified by the prediction rationale for 1-ethyl-2,3-dimethylimidazol-3-ium bis(trifluoromethanesulfonyl)amide, showcasing the specific contribution of each feature, and the sum of these contributions is exactly the predicted value. Again, the local interpretation of C-MF-based model is more easily understood than that of B-MF-based model. However, achieving global interpretations for MG- and MI-based models is more challenging. **Figures S4e and S4f** offer local interpretations for MG-based and MI-based models, in which the edge color and thickness in MG signify the significance of atoms or bonds, while pivotal prediction areas are accentuated in MI. Predominantly, ILs with fluor groups on the anions and N atom on the cations display enhanced $CO_2$ affinity, a phenomenon accurately captured by the models.[20-23] Notably, even if MD-based and MF-based models might lag in predictive performance compared to MG-based and MI-based

models, their global interpretations provide valuable insights into how specific features generally impact the target. Such knowledge is invaluable for the design or selection of candidate samples. This approach informed our recent endeavor to screen monomers for high salt rejection membrane synthesis, leveraging global interpretation insights.[17]

**Text S8.** Local interpretations of C-MF-based models for viscosity, toxicity, refractive index, and $CO_2$ absorption capacity

The effect of top-8 atom groups on ILs' properties was listed in Tabel S3. For viscosity, the alkyl chain and -OH can increase the ILs' viscosity while the -F atom and -CN group in anion can decrease its viscosity. This is consistent with previous experimental results.[24] The presence of a longer alkyl chain in the cation part of an IL tends to increase the viscosity. This is because longer chains can lead to greater van der Waals forces and entanglement between the chains, thus increasing the resistance to flow. -OH group can increase viscosity because it can form hydrogen bonds with other molecules, including water and other -OH groups, which can add to the cohesive forces in the liquid, thereby increasing its viscosity. Both -F and -CN are electron-withdrawing groups that can reduce the ability of the anion to participate in strong interactions like hydrogen bonding with the cation or other molecules, thus decreasing the viscosity of ILs. This reduced interaction generally leads to a lower viscosity, as there is less molecular interlocking impeding flow. For ILs' refractive index, the presence of -F or $-CH_3$ groups

in the anion typically decreases the refractive index of ILs. These groups tend to be less polarizable compared to others, contributing to a lower overall refractive index.[25] Aromatic carbons in the cation, due to their high degree of conjugation and polarizability, generally increase the refractive index. This is because aromatic rings can enhance electron cloud distortion under an electric field, which increases the material's refractive index.[26] For toxicity, it is essential to highlight an inverse relationship regarding toxicity and $logEC_{50}$ values. Atom groups known to increase $logEC_{50}$ values should decrease the overall toxicity of the ILs, and vice versa. Our findings align with established literature, particularly regarding the role of alkyl chains in cations,[27] and fluorine atoms in anions in augmenting the toxicity of ILs.[28, 29] For $CO_2$ absorption dataset, the identified cations predominantly consist of alkyl chains, which have been experimentally validated to increase the $CO_2$ solubility by reducing the density of ILs.[30-32] Additionally, ILs containing anions with an increased number of fluor groups demonstrate enhanced $CO_2$ solubility.[20-23]

**Text S9. The rationale for selecting non-ILs to evaluate efficacy of RU and MU**

The conventional approach involves leveraging MU and RU to filter out unreliable predictions among the candidate ILs, preserving only the reliable ones. These predictions would then be validated through experimental tests. However, given the constraints of time and resources, this methodology is labor-intensive and restrictive in scope. As an alternative, we employed non-ILs as the evaluation candidates. Given that our models

were trained exclusively on IL datasets, they are inherently ill-equipped to offer reliable predictions for non-ILs. This notion is analogous to the fallacy of using a "cat-dog" image classifier to categorize a "car" image. However, this method is a more stringent test of the MU and RU capabilities because there is a higher degree of similarity between non-ILs and ILs than between images of cat-dogs and cars; the only difference being whether or not they are charged.

**Text S10. The rationale for selecting Threshold II**

Using the threshold I and II, we quantified the unreliable predictions identified by MU and RU across these 10,000 non-ILs. If the prediction uncertainty is over the threshold values, they were deemed unreliable predictions. Both MU and RU yielded fewer unreliable predictions under Threshold I due to its relative high value (**Figure 2a**), which is far away from the ground truth. Instead, the more conservative Threshold II excluded a greater number of unreliable predictions. As previously mentioned, Threshold II designates only the majority and not the entirety of training samples as reliable, which might be more reasonable considering potential noise in some training data. Furthermore, Threshold II also amplified the efficacy of traditional MU. Therefore, we contend that Threshold II offers a more rational benchmark, and as a result, was adopted as the threshold value across all datasets and model types.

**Text S11. The details of screening process**

Our objective was to identify ILs characterized by low viscosity, low toxicity, and high $CO_2$ absorption capacity. The toxicity of ILs is a critical factor, especially considering the possibility of their accidental release into the environment. Where relevant, we standardized conditions by setting ambient pressure at 101.325 kPa and maintaining a room temperature of 298.15 K for all candidates.

We balance these trade-offs by designing a sequential screening process. As shown in Figure 2d, viscosity is given top priority because a liquid IL can easily replace amine solutions without requiring significant updates to the existing 'amine solution $CO_2$ capture technology' facilities. Next, $CO_2$ absorption capacity is prioritized over toxicity since the primary application of ILs is for $CO_2$ absorption. The toxicity of ILs becomes a priority mainly in the event of environmental leakage, which can be preemptively managed. Additionally, we have technologies to address any potential leaks, such as adsorption. Therefore, we set $CO_2$ absorption capacity as a higher priority than toxicity. This approach allows us to identify ILs with low viscosity, high $CO_2$ absorption capacity, and low toxicity

We first prioritized viscosity in our screening process, recognizing that low viscosity is essential for an IL to serve as a viable replacement for amine solutions in practical applications. Applying the RU method, 966 ILs were left. These were subsequently filtered based on a criterion of a predicted logη under 2.84 (~700 cP), yielding 966 ILs—lower than that of dual amino-functionalized ILs. These 996 ILs then

served as candidates for $CO_2$ absorption capacity assessment. Of these 996 IL candidates, 793 ILs met our RU criteria. From these, we retained 136 ILs that showcased a predicted $CO_2$ absorption capacity exceeding 0.1. For toxicity, 52 ILs were left after RU screening. A further filtration based on a predicted $\log EC_{50}$ over 1.0 left us with 37 ILs. These ILs are anticipated to be low viscosity, low toxicity, and high $CO_2$ absorption capacity. For a detailed breakdown, the SMILES representations of these 37 ILs, along with their predicted toxicity, viscosity, and $CO_2$ absorption capacity, are available in "*final_screened_results.xlsx*" file in SI.

**Table S1**. The summary of datasets used in this study.

| Dataset | Representation | Sample size | No. of ILs | No. of Cations | No. of anions | Conditions |
|---|---|---|---|---|---|---|
| Viscosity | $\log\eta$/cP | 14102 | 918 | 425 | 165 | T; P |
| Refractive index | $n_D$ | 3964 | 558 | 281 | 114 | T |
| Toxicity | $\log EC_{50}$ | 312 | 312 | 141 | 49 | — |
| $CO_2$ absorption capacity | mole fraction | 14054 | 203 | 97 | 63 | T; P |

Note: No specific criteria were used for selecting ILs in the datasets. Our approach prioritized gathering as many data points as possible to develop robust machine learning models. While the ILThermo database includes data on the refractive index, it does not provide toxicity data. Since the refractive index was not a priority property for screening ILs' $CO_2$ absorption capacity, we compiled this data from previous studies to test the RU approach.

**Table S2.** The predictive performance of models developed by different molecular representations for each dataset

| Dataset | Representation | ML algorithm | Train | | | Test | | |
|---|---|---|---|---|---|---|---|---|
| | | | RMSE | MAE | $R^2$ | RMSE | MAE | $R^2$ |
| Viscosity | B-MF | CatBoost | 0.584 | 0.434 | 0.871 | 0.699 | 0.490 | 0.816 |
| | C-MF | CatBoost | 0.434 | 0.299 | 0.929 | 0.614 | 0.408 | 0.858 |
| | MD | CatBoost | 0.270 | 0.177 | 0.972 | 0.542 | 0.353 | 0.889 |
| | MI | DN121 | 0.183 | 0.110 | 0.987 | 0.526 | 0.288 | 0.896 |
| | MG | GCN | 0.295 | 0.205 | 0.967 | 0.559 | 0.353 | 0.882 |
| | | Ensemble | ***0.253*** | ***0.165*** | ***0.976*** | ***0.500*** | ***0.304*** | ***0.906*** |
| Refractive index | B-MF | CatBoost | 0.008 | 0.005 | 0.965 | 0.012 | 0.009 | 0.910 |
| | C-MF | CatBoost | 0.006 | 0.004 | 0.978 | 0.011 | 0.007 | 0.927 |
| | MD | CatBoost | 0.006 | 0.004 | 0.980 | 0.013 | 0.009 | 0.890 |
| | MI | DN121 | 0.011 | 0.008 | 0.927 | 0.014 | 0.009 | 0.888 |
| | MG | GCN | 0.010 | 0.007 | 0.941 | 0.012 | 0.009 | 0.918 |
| | | Ensemble | ***0.007*** | ***0.005*** | ***0.972*** | ***0.011*** | ***0.008*** | ***0.931*** |
| Toxicity | B-MF | CatBoost | 0.494 | 0.331 | 0.793 | 0.546 | 0.392 | 0.631 |
| | C-MF | CatBoost | ***0.239*** | ***0.181*** | ***0.952*** | ***0.338*** | ***0.230*** | ***0.859*** |
| | MD | CatBoost | 0.280 | 0.206 | 0.933 | 0.444 | 0.310 | 0.756 |
| | MI | DN121 | 0.179 | 0.139 | 0.973 | 0.410 | 0.317 | 0.792 |
| | MG | GCN | 0.358 | 0.282 | 0.891 | 0.465 | 0.362 | 0.732 |
| | | Ensemble | 0.232 | 0.179 | 0.954 | 0.362 | 0.269 | 0.837 |
| $CO_2$ absorption | B-MF | CatBoost | 0.048 | 0.033 | 0.958 | 0.065 | 0.048 | 0.912 |
| | C-MF | CatBoost | 0.045 | 0.029 | 0.964 | 0.055 | 0.039 | 0.935 |
| | MD | CatBoost | 0.049 | 0.032 | 0.956 | 0.061 | 0.043 | 0.922 |
| | MI | DN121 | 0.045 | 0.028 | 0.964 | 0.054 | 0.038 | 0.939 |
| | MG | GCN | 0.061 | 0.044 | 0.933 | 0.066 | 0.048 | 0.907 |
| | | Ensemble | ***0.046*** | ***0.029*** | ***0.962*** | ***0.053*** | ***0.037*** | ***0.941*** |

**Table S3**. The effect of atom groups on ILs' properties revealed by the interpretation of C-MF-based models.

| | Top-1 | Top-2 | Top-3 | Top-4 | Top-5 | Top-6 | Top-7 | Top-8 |
|---|---|---|---|---|---|---|---|---|
| Viscosity | | | | | | | | |
| Atom groups | 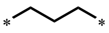 | 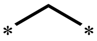 | 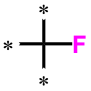 | $Cl^-$ | 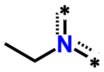 | 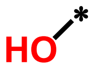 | 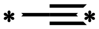 | 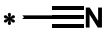 |
| Effect | ↑ | ↑ | ↓ | ↑ | ↓ | ↑ | ↓ | ↓ |
| Source | Cation | Cation | Anion | Anion | Cation | Cation | Anion | Anion |
| Refractive index | | | | | | | | |

| | | | | | | | | |
|---|---|---|---|---|---|---|---|---|
| Atom groups | 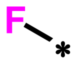 | 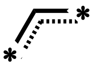 | 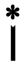 | 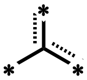 | 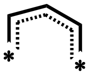 | 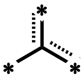 | 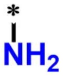 | 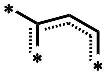 |
| Effect | ↓ | ↑ | ↓ | ↑ | ↑ | ↑ | ↑ | ↑ |
| Source | Anion | Cation | Anion | Cation | Cation | Anion | Anion | Anion |
| Toxicity | | | | | | | | |
| Atom groups | 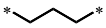 | 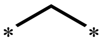 | 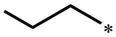 | 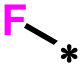 | 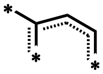 | $Cl^-$ | 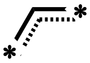 | 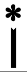 |
| Effect | ↓ | ↓ | ↓ | ↓ | ↓ | ↑ | ↓ | ↓ |
| Source | Cation | Cation | Cation | Anion | Cation | Anion | Cation | Cation |
| $CO_2$ absorption | | | | | | | | |
| Atom groups | 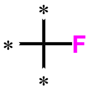 | 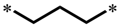 | 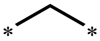 | 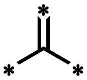 | 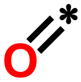 | 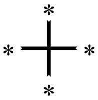 | 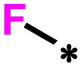 | 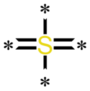 |
| Effect | ↑ | ↑ | ↑ | ↑ | ↑ | ↑ | ↑ | ↑ |
| Source | Anion | Cation | Cation | Anion | Anion | Anion | Anion | Anion |

**Table S4**. The training and test performance of the other three MF-based, MD-based and MG-based models for each dataset, respectively.

| Dataset | RP | HP | RMSE Train | MAE Train | $R^2$ Train | RMSE Test | MAE Test | $R^2$ Test |
|---|---|---|---|---|---|---|---|---|
| Viscosity | MF | 2nd best | 0.486 | 0.339 | 0.911 | 0.631 | 0.434 | 0.850 |
| | | 3rd best | 0.496 | 0.348 | 0.907 | 0.640 | 0.446 | 0.846 |
| | | 4th best | 0.515 | 0.359 | 0.900 | 0.648 | 0.449 | 0.842 |
| | MD | 2nd best | 0.260 | 0.172 | 0.974 | 0.525 | 0.344 | 0.896 |
| | | 3rd best | 0.322 | 0.213 | 0.961 | 0.573 | 0.369 | 0.876 |
| | | 4th best | 0.329 | 0.216 | 0.959 | 0.563 | 0.364 | 0.880 |
| | MG | 2nd best | 0.755 | 0.548 | 0.785 | 0.785 | 0.554 | 0.768 |
| | | 3rd best | 0.694 | 0.474 | 0.818 | 0.741 | 0.498 | 0.793 |
| | | 4th best | 0.613 | 0.442 | 0.859 | 0.686 | 0.480 | 0.823 |
| Refractive index | MF | 2nd best | 0.007 | 0.005 | 0.968 | 0.013 | 0.009 | 0.893 |
| | | 3rd best | 0.007 | 0.005 | 0.968 | 0.013 | 0.008 | 0.901 |
| | | 4th best | 0.008 | 0.006 | 0.962 | 0.012 | 0.008 | 0.914 |
| | MD | 2nd best | 0.004 | 0.002 | 0.992 | 0.012 | 0.008 | 0.918 |

| | | | | | | | | |
|---|---|---|---|---|---|---|---|---|
| | | 3rd best | 0.008 | 0.005 | 0.963 | 0.013 | 0.009 | 0.893 |
| | | 4th best | 0.008 | 0.005 | 0.963 | 0.014 | 0.009 | 0.873 |
| | MG | 2nd best | 0.010 | 0.007 | 0.939 | 0.012 | 0.009 | 0.913 |
| | | 3rd best | 0.010 | 0.007 | 0.935 | 0.012 | 0.010 | 0.910 |
| | | 4th best | 0.010 | 0.007 | 0.933 | 0.013 | 0.010 | 0.902 |
| Toxicity | MF | 2nd best | 0.277 | 0.213 | 0.935 | 0.319 | 0.224 | 0.874 |
| | | 3rd best | 0.288 | 0.221 | 0.929 | 0.320 | 0.221 | 0.873 |
| | | 4th best | 0.294 | 0.222 | 0.927 | 0.342 | 0.234 | 0.855 |
| | MD | 2nd best | 0.273 | 0.201 | 0.937 | 0.434 | 0.306 | 0.766 |
| | | 3rd best | 0.297 | 0.222 | 0.925 | 0.444 | 0.306 | 0.756 |
| | | 4th best | 0.302 | 0.228 | 0.923 | 0.417 | 0.300 | 0.785 |
| | MG | 2nd best | 0.253 | 0.190 | 0.946 | 0.546 | 0.385 | 0.631 |
| | | 3rd best | 0.250 | 0.188 | 0.947 | 0.489 | 0.368 | 0.704 |
| | | 4th best | 0.412 | 0.325 | 0.856 | 0.578 | 0.405 | 0.585 |
| $CO_2$ absorption | MF | 2nd best | 0.041 | 0.026 | 0.970 | 0.054 | 0.038 | 0.938 |
| | | 3rd best | 0.048 | 0.032 | 0.959 | 0.057 | 0.040 | 0.932 |
| | | 4th best | 0.045 | 0.030 | 0.963 | 0.056 | 0.040 | 0.933 |
| | MD | 2nd best | 0.048 | 0.031 | 0.958 | 0.062 | 0.042 | 0.920 |
| | | 3rd best | 0.049 | 0.032 | 0.957 | 0.061 | 0.043 | 0.921 |
| | | 4th best | 0.050 | 0.033 | 0.955 | 0.060 | 0.042 | 0.924 |
| | MG | 2nd best | 0.075 | 0.057 | 0.900 | 0.072 | 0.056 | 0.892 |
| | | 3rd best | 0.084 | 0.064 | 0.875 | 0.087 | 0.068 | 0.840 |
| | | 4th best | 0.086 | 0.067 | 0.869 | 0.090 | 0.071 | 0.830 |

**Table S5.** The number of unreliable predictions identified by each type of uncertainty for 5 groups of random selected 10,000 non-ILs

| Dataset | Groups | MF-MU | MD-MU | MG-MU | RU |
|---|---|---|---|---|---|
| Viscosity | G1 | 9419 | 9862 | 7410 | 9993 |
| | G2 | 9341 | 9785 | 7762 | 9974 |
| | G3 | 9398 | 9874 | 7694 | 9998 |

| | | | | | |
|---|---|---|---|---|---|
| | G4 | 9029 | 9788 | 7463 | 9998 |
| | G5 | 9413 | 9753 | 7378 | 9989 |
| Refractive index | G1 | 7914 | 8545 | 7882 | 9243 |
| | G2 | 8025 | 8624 | 8237 | 9193 |
| | G3 | 7992 | 8406 | 7471 | 9177 |
| | G4 | 7942 | 7880 | 7932 | 9215 |
| | G5 | 7571 | 8085 | 7416 | 9136 |
| Toxicity | G1 | 5026 | 8892 | 9608 | 9913 |
| | G2 | 4557 | 9342 | 9463 | 9926 |
| | G3 | 5213 | 9407 | 9145 | 9961 |
| | G4 | 5068 | 9220 | 9425 | 9949 |
| | G5 | 4612 | 9356 | 9510 | 9896 |
| $CO_2$ absorption | G1 | 6770 | 3938 | 3816 | 9109 |
| | G2 | 7144 | 4741 | 3871 | 9170 |
| | G3 | 6360 | 4629 | 3494 | 9202 |
| | G4 | 6013 | 4951 | 3315 | 9046 |
| | G5 | 6678 | 4039 | 3034 | 9397 |

**Table S6**. The node features for each dataset

| Dataset | Atom feature | size | Description |
|---|---|---|---|
| Viscosity | atom type | 12 | [O, P, B, I, Sb, C, N, Cl, As, S, F] (one-hot) |
| Refractive index | | 11 | [S, C, O, H, F, Cl, I, Br, P, N, B] (one-hot) |
| Toxicity | | 11 | [Br, F, I, O, S, H, B, N, C, P, Cl] (one-hot) |
| $CO_2$ adsorption | | 10 | [N, F, C, P, Cl, Br, S, B, O, H] (one-hot) |
| All | degree | 6 | Number of covalent bonds [0,1,2,3,4,5] (one-hot) |
| All | formal charge | 1 | Electrical charge (integer) |
| All | radical electrons | 1 | Number of radical electrons (integer) |
| All | hybridization | 6 | [sp, $sp^2$, $sp^3$, $sp^3d$, $sp^3d^2$, other] (one-hot) |
| All | Aromaticity | 1 | whether the atom is part of an aromatic system [0/1] (one-hot) |
| All | hydrogens | 5 | number of connected hydrogens (integer) |
| All | chirality | 1 | whether the atom is chiral center [0/1] (one-hot) |

| | | | |
|---|---|---|---|
| All | Chirality type | 2 | [R, S] (one-hot) |
| Viscosity, Refractive index, $CO_2$ adsorption | Temperature | 1 | Experimental condition (float) |
| Viscosity, $CO_2$ adsorption | Pressure | 1 | Experimental condition (float) |

**Table S7**. The 9 candidate ML algorithms

| Linear | Non-linear |
|---|---|
| Lasso and Ridge regression | CatBoostRegressor, XGBRegressor, SVM, RandomForestRegressor, AdaBoostRegressor, BaggingRegressor, GradientBoostingRegressor |

**Table S8**. The final number of descriptors and their names used developing MD-based models for each datasets

| Dataset | MDs |
|---|---|
| Viscosity | **159 features**: 'BCUTdv-1l_A', 'RPCG_C', 'ATSC1dv_A', 'GGI5_C', 'ETA_dBeta_A', 'PEOE_VSA4_A', 'ATSC3p_C', 'ATSC4Z_A', 'ATSC4dv_A', 'Pressure, kPa', 'ATSC3c_C', 'AATSC0i_A', 'BCUTs-1h_C', 'BCUTd-1h_C', 'Xch-5dv_C', 'AATS0p_A', 'ATSC3pe_A', 'ATSC1pe_A', 'ATSC1dv_C', 'ATSC5Z_A', 'GATS1s_C', 'ATSC2s_C', 'SlogP_VSA8_A', 'SlogP_VSA1_A', 'BCUTpe-1l_A', 'MATS2s_C', 'BCUTd-1l_A', 'Xp-7d_A', 'ATSC0Z_A', 'GATS1Z_C', 'ETA_shape_x_A', 'ATSC8d_A', 'RNCG_C', 'ATSC0p_A', 'MATS1s_C', 'PEOE_VSA5_A', 'ATSC2pe_A', 'ATSC4d_A', 'SIC0_C', 'MATS2c_C', 'EState_VSA2_A', 'RotRatio_C', 'FilterItLogS_C', 'ETA_eta_F_C', 'fMF_C', 'SpMAD_A_A', 'ATSC3Z_C', 'BCUTpe-1h_A', 'FilterItLogS_A', 'BertzCT_C', 'SpMax_A_C', 'JGI3_C', 'JGI3_A', 'JGI5_C', 'ATSC5dv_C', 'AATSC0dv_A', 'ATSC0s_C', 'BCUTv-1h_A', 'ATSC1Z_A', 'ATSC7dv_C', 'ATSC1Z_C', 'VR1_A_C', 'ATS0Z_A', 'ETA_beta_ns_A', 'GGI7_C', 'BCUTi-1l_A', 'AATSC1p_C', 'BCUTi-1h_C', 'ATSC2dv_A', 'BCUTse-1h_C', 'ATSC5c_C', 'VSA_EState3_C', 'ATSC7d_C', 'ATSC3v_A', 'AMID_C_C', 'VSA_EState8_C', 'PEOE_VSA9_A', 'ATS0dv_C', 'nC_A', 'VE2_A_C', 'ATSC3dv_C', 'ATSC4c_C', 'ATSC7s_C', 'EState_VSA4_C', 'PEOE_VSA9_C', 'AATS0Z_C', 'AETA_beta_A', 'Temperature, K', 'fragCpx_C', 'AMID_N_C', 'ATSC2d_A', 'ATSC0c_C', 'JGI6_C', 'PEOE_VSA10_C', 'AMID_C_A', 'IC4_C', 'GATS2se_C', 'BCUTc-1l_C', 'ATSC6c_C', 'ATSC5i_A', 'AATS0v_A', 'Xpc-4d_C', 'AATSC1s_C', 'BCUTZ-1h_A', 'AATSC1Z_C', 'JGI7_C', 'AATSC1pe_C', 'PEOE_VSA1_A', 'ATSC3v_C', 'ATS0p_A', 'ETA_shape_p_A', 'IC1_A', 'SlogP_VSA2_A', 'BCUTd-1h_A', 'ATSC1d_A', 'BCUTZ-1h_C', 'ATSC0dv_A', 'nBase_C', 'ATSC1i_A', 'ATSC2v_A', 'ATSC8c_C', 'Xc-3d_C', 'ATSC7se_C', 'piPC7_C', 'GGI4_C', 'TopoShapeIndex_C', 'ATSC8dv_C', 'ATSC2d_C', 'piPC7_A', 'AATSC1se_C', 'ATSC5d_A', 'SpDiam_A_C', 'ATSC1s_C', 'AATSC1d_C', 'BCUTd-1l_C', 'VSA_EState2_C', 'BCUTs-1l_C', 'AATS0dv_A', 'AATSC0v_A', 'IC0_A', 'CIC0_A', 'BCUTp-1h_A', 'ATSC2Z_A', 'nBondsM_A', 'ATSC3dv_A', 'ATSC2p_A', 'MIC1_C', 'nN_A', 'n6ARing_C', 'ATSC5Z_C', 'SdssC_A', 'AATSC0Z_A', 'GATS1p_C', 'AATSC2c_C', 'GATS2s_C', 'SLogP_A', 'AATSC1v_C', 'SpAbs_A_C', 'AETA_eta_L_A'] |

| | |
|---|---|
| | 298 attributes confirmed unimportant: ['NsCH3_A', 'AATS0i_C', 'NdssC_C', 'NddsN_A', 'n10FARing_C', 'VSA_EState6_A', 'ATSC6v_A', 'VSA_EState9_A', 'Lipinski_C', 'PEOE_VSA11_A', 'BCUTZ-1l_C', 'Xc-3dv_C', 'nBase_A', 'EState_VSA9_A', 'nAcid_C', 'AATSC1dv_C', 'n9FRing_C', 'fragCpx_A', 'nARing_C', 'SsssCH_A', 'n10FRing_C', 'AETA_beta_s_C', 'n6ARing_A', 'nHetero_C', 'NsssB_A', 'NdO_C', 'SlogP_VSA11_C', 'NssO_C', 'AATSC2s_C', 'NdCH2_C', 'NssssN_C', 'NddssS_C', 'ATS7Z_A', 'ATSC8pe_A', 'C1SP3_A', 'AMID_X_A', 'PEOE_VSA2_C', 'AATS0p_C', 'SssssC_C', 'nP_C', 'nRing_C', 'ATSC8p_A', 'ATSC8Z_A', 'NddC_A', 'PEOE_VSA5_C', 'AATSC0d_C', 'ATSC3d_C', 'AATS1dv_C', 'ATSC5p_C', 'BCUTc-1h_C', 'GhoseFilter_A', 'n7Ring_C', 'PEOE_VSA7_A', 'ETA_dAlpha_A_C', 'Xc-4dv_A', 'AETA_eta_RL_C', 'nAromAtom_A', 'PEOE_VSA13_A', 'nHBDon_C', 'AATSC0p_C', 'nARing_A', 'EState_VSA3_C', 'GATS2Z_C', 'nBondsT_A', 'EState_VSA3_A', 'IC0_C', 'NdsN_A', 'NsNH2_C', 'C1SP2_A', 'nRot_A', 'nB_A', 'ETA_dEpsilon_C_C', 'ATSC3d_A', 'JGI10_A', 'ATSC4i_C', 'AETA_eta_F_C', 'nS_C', 'Xch-7dv_C', 'nAcid_A', 'SlogP_VSA11_A', 'AATSC0dv_C', 'NaasC_C', 'NssCH2_A', 'NsCH3_C', 'AETA_alpha_C', 'PEOE_VSA12_A', 'SlogP_VSA7_C', 'NssNH_A', 'AETA_beta_C', 'EState_VSA9_C', 'PEOE_VSA1_C', 'ATSC8i_A', 'ATSC7v_C', 'ATSC1p_A', 'NsOH_C', 'EState_VSA1_C', 'ATSC8i_C', 'GATS2c_C', 'TopoPSA(NO)_C', 'AATS0d_C', 'GATS1v_C', 'NsssCH_C', 'ATSC7dv_A', 'AETA_eta_B_C', 'NssNH2_C', 'n5ARing_A', 'ATSC8v_C', 'SdsCH_A', 'JGI10_C', 'ETA_dBeta_C', 'JGI8_A', 'n6HRing_A', 'AATSC2dv_C', 'SpMax_A_A', 'nCl_C', 'EState_VSA5_A', 'NaasC_A', 'piPC10_A', 'VSA_EState5_A', 'n6aHRing_A', 'AATS2d_C', 'nI_A', 'ATS6d_A', 'AATSC2Z_C', 'SM1_DzZ_C', 'SMR_VSA3_A', 'n6AHRing_A', 'NssssN_A', 'NsNH2_A', 'SlogP_VSA7_A', 'JGI6_A', 'NdsCH_C', 'SsssCH_C', 'C2SP3_A', 'nHBAcc_C', 'C1SP3_C', 'NdssC_A', 'EState_VSA8_A', 'SMR_VSA6_C', 'Xc-4d_A', 'AMID_O_C', 'ATSC6d_C', 'nO_C', 'PEOE_VSA13_C', 'ATSC7i_A', 'NaaaC_C', 'EState_VSA7_A', 'nAromAtom_C', 'ATSC7d_A', 'SMR_VSA6_A', 'NaaN_C', 'EState_VSA4_A', 'NsNH3_C', 'ATSC5v_A', 'AXp-0d_C', 'GGI10_A', 'NaaaC_A', 'JGI5_A', 'piPC9_A', 'VSA_EState8_A', 'SMR_VSA4_C', 'ATSC4v_A', 'NsssNH_A', 'Xch-7d_A', 'NssO_A', 'NdsssP_A', 'Xch-7dv_A', 'C3SP3_A', 'GGI9_A', 'Xc-6dv_C', 'VR1_A_A', 'AATS1i_C', 'JGI9_C', 'ATSC5v_C', 'NsBr_A', 'AATS2p_C', 'n5ARing_C', 'NdCH2_A', 'nFARing_C', 'SssNH_A', 'nP_A', 'ETA_beta_ns_d_C', 'VSA_EState5_C', 'Xch-6d_A', 'ETA_shape_y_C', 'nHRing_A', 'nHBDon_A', 'ATSC1v_C', 'ETA_epsilon_3_C', 'ATSC1d_C', 'SsssB_A', 'SlogP_VSA5_A', 'AMID_O_A', 'Lipinski_A', 'VSA_EState7_A', 'nHetero_A', 'fMF_A', 'ATSC7v_A', 'NaasN_A', 'nBr_C', 'ATSC6v_C', 'SlogP_VSA8_C', 'ATSC8dv_A', 'PEOE_VSA8_A', 'GATS2dv_C', 'BCUTdv-1h_A', 'JGI9_A', 'Xch-7d_C', 'NdNH_C', 'SMR_VSA7_A', 'HybRatio_C', 'ETA_dEpsilon_D_C', 'NaaS_C', 'MATS2dv_C', 'Xch-6dv_A', 'AXp-1d_C', 'NaaNH_C', 'SlogP_VSA4_C', 'ETA_dEpsilon_B_A', 'AATSC2d_C', 'EState_VSA6_A', 'n6Ring_C', 'ATSC5d_C', 'nF_C', 'JGI7_A', 'ATSC7p_A', 'ATSC6Z_A', 'nF_A', 'ATSC7Z_C', 'ATSC2Z_C', 'piPC9_C', 'GGI3_C', 'ATSC5dv_A', 'NssNH_C', 'NdsCH_A', 'NsNH3_A', 'C2SP2_C', 'C2SP2_A', 'PEOE_VSA10_A', 'Xc-5dv_C', 'GhoseFilter_C', 'SlogP_VSA1_C', 'PEOE_VSA6_A', 'NdsN_C', 'NaaN_A', 'Xc-5dv_A', 'NsssN_C', 'C3SP3_C', 'ETA_shape_y_A', 'ATSC6p_A', 'NaaNH_A', 'AATS0v_C', 'C3SP2_C', 'nBondsD_C', 'ATSC8v_A', 'BCUTse-1l_C', 'SddssS_C', 'SdssC_C', 'NssS_A', 'AATS1p_C', 'nAtom_A', 'ATS7dv_A', 'ATSC3p_A', 'C1SP1_A', 'SssssB_A', 'ETA_dEpsilon_B_C', 'Xch-5dv_A', 'NssS_C', 'NsI_A', 'PEOE_VSA11_C', 'n6Ring_A', 'PEOE_VSA7_C', 'SMR_VSA4_A', 'nCl_A', 'NsssN_A', 'piPC8_A', 'EState_VSA2_C', 'VE2_A_A', 'nN_C', 'ATSC5i_C', 'ETA_beta_ns_d_A', 'ETA_dAlpha_B_C', 'nBr_A', 'C3SP2_A', 'JGI2_C', 'Xch-5d_A', 'SddsN_A', 'PEOE_VSA12_C', 'NaasN_C', 'ATSC8Z_C', 'VSA_EState4_C', 'C1SP2_C', 'ETA_shape_x_C', 'ATSC6dv_A', 'ATSC7p_C', 'C4SP3_A', 'ATSC1c_C', 'nH_A' |
| Refractive index | **124 features**: 'VSA_EState7_A', 'n6Ring_C', 'GATS1v_C', 'GATS1p_C', 'BCUTd-1l_C', 'AATS0dv_A', 'ATSC3i_A', 'EState_VSA8_A', 'ATSC2i_A', 'fragCpx_C', 'ATSC3Z_C', 'BCUTi-1h_C', 'ATSC7dv_C', 'AATSC3Z_C', 'ETA_eta_F_C', 'AATS3d_C', 'nBase_A', 'Xch-6d_A', 'ATSC1Z_A', 'AATS0Z_A', 'ATSC7s_C', 'AATSC0Z_A', 'PEOE_VSA2_A', 'nHeavyAtom_A', 'GATS2v_C', 'GATS1s_C', 'Xch-7d_A', 'EState_VSA9_A', 'ATSC3p_A', 'AATS0se_A', 'ATSC2d_A', 'ATSC3se_A', 'AATS4Z_C', 'SlogP_VSA4_C', 'ATSC1p_C', 'AATS4i_C', |

| | |
|---|---|
| | 'Temperature', 'AATS0d_A', 'MIC0_A', 'AATS2i_C', 'ATSC1p_A', 'ATSC1d_A', 'AATS3Z_C', 'AATSC1p_C', 'BCUTZ-1l_C', 'BCUTse-1l_C', 'ATSC3v_A', 'AATSC0d_C', 'VSA_EState9_A', 'AXp-0dv_A', 'AATSC1v_C', 'NsNH2_A', 'ATSC1v_A', 'AATS4d_C', 'PEOE_VSA10_C', 'PEOE_VSA1_A', 'ATSC5se_C', 'ATS0dv_C', 'AATS0v_C', 'AATS3v_C', 'MATS1s_C', 'ATS1dv_A', 'MATS2dv_C', 'nHetero_A', 'VSA_EState4_A', 'VR1_A_C', 'nF_A', 'MATS4c_C', 'AATS0i_C', 'BCUTd-1h_C', 'ATSC8c_C', 'ATS0Z_A', 'VSA_EState3_C', 'ATSC2Z_C', 'ATSC1i_A', 'AXp-1dv_C', 'SLogP_A', 'AATS3dv_C', 'Xc-4d_A', 'GATS1d_C', 'AATSC0p_A', 'AATS1p_C', 'JGI4_A', 'ATSC1pe_C', 'nAromAtom_C', 'SlogP_VSA2_A', 'ATSC1se_A', 'AATS0d_C', 'RPCG_C', 'piPC7_C', 'VSA_EState1_C', 'CIC1_A', 'BalabanJ_A', 'MATS3s_C', 'AATSC0dv_A', 'ATSC2Z_A', 'PEOE_VSA11_C', 'ATSC2p_A', 'AATS4dv_C', 'ATSC3v_C', 'IC3_C', 'ATSC1d_C', 'JGI1_A', 'NsCH3_A', 'ETA_epsilon_5_C', 'Xch-7dv_C', 'IC1_A', 'ATSC1s_C', 'C2SP2_C', 'AATS0v_A', 'MATS2c_C', 'SMR_VSA6_A', 'ATSC5v_A', 'AXp-0d_C', 'SlogP_VSA1_A', 'AATS3i_C', 'FilterItLogS_A', 'AATSC0i_C', 'IC0_A', 'AATSC0se_A', 'BCUTc-1l_C', 'AATSC0c_C', 'ATSC2dv_A', 'ATS3dv_C' |
| Toxicity | **20 features**: 'AATS0s_C', 'SpMax_A_A', 'SpAbs_A_C', 'AATSC3Z_C', 'ATS4dv_A', 'GGI7_C', 'FilterItLogS_C', 'fragCpx_C', 'VSA_EState7_C', 'SIC0_C', 'EState_VSA8_C', 'ETA_dBeta_A', 'ATSC3Z_C', 'RNCG_C', 'AETA_alpha_C', 'ATSC1dv_C', 'FilterItLogS_A', 'GGI8_C', 'VE2_A_C', 'EState_VSA9_C' |
| $CO_2$ absorption | **47 features**: 'ETA_dEpsilon_D_A', 'ATSC2c_C', 'IC3_C', 'ETA_beta_ns_A', 'MATS2c_C', 'ATSC5d_A', 'VE2_A_C', 'ATSC5se_A', 'ATSC8c_C', 'ATSC2d_A', 'T', 'ETA_dBeta_C', 'ATSC1p_A', 'BCUTdv-1l_C', 'nC_A', 'SpMax_A_A', 'BCUTZ-1h_A', 'BCUTse-1l_A', 'JGI9_C', 'AMID_O_A', 'VSA_EState4_A', 'GATS4p_C', 'ATSC1Z_A', 'SLogP_A', 'SpAbs_A_A', 'FilterItLogS_A', 'AMID_A', 'AATSC0d_C', 'SlogP_VSA1_A', 'VR1_A_A', 'ATSC1dv_A', 'SM1_Dzv_A', 'AATS0Z_A', 'ATSC2p_A', 'CIC1_A', 'ATSC2Z_C', 'MATS4i_C', 'VE2_A_A', 'PEOE_VSA1_A', 'ATSC3i_A', 'AATSC0Z_A', 'P', 'ATSC1d_C', 'AATS0d_A', 'SpAbs_A_C', 'ETA_shape_y_C', 'JGI3_C' |

**Table S9**. The candidate hyperparameters of CatBoost for MF and MD

| Name | Range |
|---|---|
| depth | (1, 6) |
| l2_leaf_reg | (3, 100) |
| learning_rate | (0.0001, 0.025) |
| iterations | (1, 1000) |
| bagging_temperature | (1, 200) |
| random_strength | (1, 200) |

**Table S10**. The candidate hyperparameters for MG

| Name | Range |
|---|---|
| Batch size | (64, 128) |
| Drop rate | (0, 0.5) |
| Hidden layer | (1, 2) |
| Hidden size | (16, 1024) |
| Learning rate | (0.0005, 0.01) |

## References

(1) Lei, X.; Xu, Y.; Zhu, L.; Wang, X. Highly efficient and reversible CO 2 capture through 1, 1, 3, 3-tetramethylguanidinium imidazole ionic liquid. *Rsc Adv* **2014**, *4* (14), 7052-7057.
(2) Moriwaki, H.; Tian, Y.-S.; Kawashita, N.; Takagi, T. Mordred: a molecular descriptor calculator. *Journal of Cheminformatics* **2018**, *10* (1), 4. DOI: 10.1186/s13321-018-0258-y.
(3) Weininger, D. SMILES, a chemical language and information system. 1. Introduction to methodology and encoding rules. *Journal of Chemical Information and Computer Sciences* **1988**, *28* (1), 31-36. DOI: 10.1021/ci00057a005.
(4) Zhong, S.; Guan, X. Count-Based Morgan Fingerprint: A More Efficient and Interpretable Molecular Representation in Developing Machine Learning-Based Predictive Regression Models for Water Contaminants' Activities and Properties. *Environmental Science & Technology* **2023**, *57* (46), 18193-18202. DOI: 10.1021/acs.est.3c02198.
(5) Zhong, S.; Zhang, K.; Wang, D.; Zhang, H. Shedding Light On "Black Box" Machine Learning Models for Predicting the Reactivity of HO• Radicals toward Organic Compounds. *Chemical Engineering Journal* **2020**, 126627. DOI: https://doi.org/10.1016/j.cej.2020.126627.
(6) Zhong, S.; Zhang, Y.; Zhang, H. Machine Learning-Assisted QSAR Models on Contaminant Reactivity Toward Four Oxidants: Combining Small Data Sets and Knowledge Transfer. *Environmental Science & Technology* **2022**, *56* (1), 681-692.
(7) Rogers, D.; Hahn, M. Extended-Connectivity Fingerprints. *Journal of Chemical Information and Modeling* **2010**, *50* (5), 742-754. DOI: 10.1021/ci100050t.
(8) Xiong, Z.; Wang, D.; Liu, X.; Zhong, F.; Wan, X.; Li, X.; Li, Z.; Luo, X.; Chen, K.; Jiang, H. Pushing the boundaries of molecular representation for drug discovery with the graph attention mechanism. *Journal of medicinal chemistry* **2019**, *63* (16), 8749-8760.
(9) Ding, Y.; Chen, M.; Guo, C.; Zhang, P.; Wang, J. Molecular fingerprint-based machine learning assisted QSAR model development for prediction of ionic liquid properties. *Journal of Molecular Liquids* **2021**, *326*, 115212.
(10) Keany, E. BorutaShap: A wrapper feature selection method which combines the Boruta feature selection algorithm with Shapley values. *Zenodo https://zenodo. org/record/4247618 (Accessed October 25, 2021)* **2020**.
(11) Zhang, S.; Tong, H.; Xu, J.; Maciejewski, R. Graph convolutional networks: a comprehensive review. *Computational Social Networks* **2019**, *6* (1), 1-23.
(12) Kipf, T. N.; Welling, M. Semi-supervised classification with graph convolutional networks. *arXiv preprint arXiv:1609.02907* **2016**.
(13) Srivastava, N.; Hinton, G.; Krizhevsky, A.; Sutskever, I.; Salakhutdinov, R. Dropout: a simple way to prevent neural networks from overfitting. *The journal of machine learning research* **2014**, *15* (1), 1929-1958.
(14) Zhou, K.; Dong, Y.; Wang, K.; Lee, W. S.; Hooi, B.; Xu, H.; Feng, J. Understanding and resolving performance degradation in graph convolutional networks. *arXiv preprint arXiv:2006.07107* **2020**.
(15) Zhong, S.; Hu, J.; Yu, X.; Zhang, H. Molecular image-convolutional neural network (CNN) assisted QSAR models for predicting contaminant reactivity toward OH radicals: Transfer learning, data augmentation and model interpretation. *Chemical Engineering Journal* **2021**, *408*, 127998.
(16) Huang, G.; Liu, Z.; Van Der Maaten, L.; Weinberger, K. Q. Densely connected convolutional networks. In *Proceedings of the IEEE conference on computer vision and pattern recognition*, 2017; pp 4700-4708.

(17) Gao, H.; Zhong, S.; Zhang, W.; Igou, T.; Berger, E.; Reid, E.; Zhao, Y.; Lambeth, D.; Gan, L.; Afolabi, M. A.; et al. Revolutionizing Membrane Design Using Machine Learning-Bayesian Optimization. *Environmental Science & Technology* **2021**. DOI: 10.1021/acs.est.1c04373.

(18) Selvaraju, R. R.; Das, A.; Vedantam, R.; Cogswell, M.; Parikh, D.; Batra, D. Grad-CAM: Why did you say that? *arXiv preprint arXiv:1611.07450* **2016**.

(19) Sundararajan, M.; Taly, A.; Yan, Q. Axiomatic attribution for deep networks. In *International Conference on Machine Learning*, 2017; PMLR: pp 3319-3328.

(20) Baltus, R. E.; Culbertson, B. H.; Dai, S.; Luo, H.; DePaoli, D. W. Low-pressure solubility of carbon dioxide in room-temperature ionic liquids measured with a quartz crystal microbalance. *The Journal of Physical Chemistry B* **2004**, *108* (2), 721-727.

(21) Zhang, X.; Liu, Z.; Wang, W. Screening of ionic liquids to capture CO2 by COSMO-RS and experiments. *AIChE J.* **2008**, *54* (10), 2717-2728.

(22) Jung, Y.-H.; Jung, J.-Y.; Jin, Y.-R.; Lee, B.-C.; Baek, I.-H.; Kim, S.-H. Solubility of carbon dioxide in imidazolium-based ionic liquids with a methanesulfonate anion. *Journal of Chemical & Engineering Data* **2012**, *57* (12), 3321-3329.

(23) Pereiro, A. B.; Tomé, L. C.; Martinho, S.; Rebelo, L. P. N.; Marrucho, I. M. Gas permeation properties of fluorinated ionic liquids. *Industrial & Engineering Chemistry Research* **2013**, *52* (14), 4994-5001.

(24) Al-Masri, D.; Yunis, R.; Hollenkamp, A. F.; Doherty, C. M.; Pringle, J. M. The influence of alkyl chain branching on the properties of pyrrolidinium-based ionic electrolytes. *Physical Chemistry Chemical Physics* **2020**, *22* (32), 18102-18113, Article. DOI: 10.1039/d0cp03046e.

(25) Fröba, A. P.; Kremer, H.; Leipertz, A. Density, Refractive Index, Interfacial Tension, and Viscosity of Ionic Liquids [EMIM][EtSO4], [EMIM][NTf2], [EMIM][N(CN)2], and [OMA][NTf2] in Dependence on Temperature at Atmospheric Pressure. *The Journal of Physical Chemistry B* **2008**, *112* (39), 12420-12430. DOI: 10.1021/jp804319a.

(26) Kayama, Y.; Ichikawa, T.; Ohno, H. Transparent and colourless room temperature ionic liquids having high refractive index over 1.60. *Chem. Commun.* **2014**, *50* (94), 14790-14792, Article. DOI: 10.1039/c4cc06145d.

(27) Kuroda, K. Simple overview of toxicity of ionic liquids and designs of biocompatible ionic liquids. *New Journal of Chemistry* **2022**.

(28) Petkovic, M.; Seddon, K. R.; Rebelo, L. P. N.; Pereira, C. S. Ionic liquids: a pathway to environmental acceptability. *Chemical Society Reviews* **2011**, *40* (3), 1383-1403.

(29) Cho, C.-W.; Pham, T. P. T.; Zhao, Y.; Stolte, S.; Yun, Y.-S. Review of the toxic effects of ionic liquids. *Sci Total Environ* **2021**, *786*, 147309.

(30) Aki, S. N.; Mellein, B. R.; Saurer, E. M.; Brennecke, J. F. High-pressure phase behavior of carbon dioxide with imidazolium-based ionic liquids. *The Journal of Physical Chemistry B* **2004**, *108* (52), 20355-20365.

(31) Yunus, N. M.; Mutalib, M. A.; Man, Z.; Bustam, M. A.; Murugesan, T. Solubility of CO2 in pyridinium based ionic liquids. *Chemical engineering journal* **2012**, *189*, 94-100.

(32) Huang, X.; Margulis, C. J.; Li, Y.; Berne, B. J. Why is the partial molar volume of CO2 so small when dissolved in a room temperature ionic liquid? Structure and dynamics of CO2 dissolved in [Bmim+][PF6-]. *Journal of the American Chemical Society* **2005**, *127* (50), 17842-17851.
